# Evidence on the impact of community health workers in the prevention, identification, and management of undernutrition amongst children under the age of five in conflict-affected or fragile settings: a systematic literature review

**DOI:** 10.1186/s13031-024-00575-8

**Published:** 2024-02-27

**Authors:** Rachel Bridge, Tracy Kuo Lin

**Affiliations:** 1 San Francisco, USA; 2 Institute for Health and Aging, Department of Social and Behavioral Sciences, University of California, San Francisco, 490 Illinois St, 123K, San Francisco, CA 94158 USA; 3 Middle East Centre, London School of Economics and Political Science, London, UK; 4 Boston, USA

**Keywords:** Children under five, Undernutrition, Conflict, Conflict-affected and fragile setting, Systematic review

## Abstract

**Background:**

Malnutrition, specifically undernutrition, is a significant global challenge that contributes to nearly half of deaths in children under the age of five. The burden of undernutrition is disproportionately borne by conflict-affected, fragile settings (CAFS); children living in a conflict zone being more than twice as likely to suffer from malnourishment. Community health worker (CHW) models have been employed in CAFS to improve healthcare coverage and identify and treat illnesses. However, there lacks systematic evidence on the impact of CHW models in preventing, identifying, and managing child undernutrition in CAFS. We conducted this review to systematically evaluate evidence of CHW models in preventing, identifying, and managing undernutrition in children under the age of five in CAFS.

**Methodology:**

This review followed the Preferred Reporting Items for Systematic Reviews and Meta-Analyses reporting standards. The search strategy was developed using the Population-Intervention-Comparisons-Outcomes-Setting framework as a guide. Searches were performed using Ovid online database search platform, searching the databases of Ovid MEDLINE(R), COCHRANE, Embase Classic, Embase, Econlit, Global Health, SCOPUS, and Social Policy and Practice. Peer-reviewed publications were eligible for inclusion if they evaluated an intervention using a CHW model that aims to prevent, identify, or manage some form of undernutrition in children under five in a CAFS.

**Results:**

We identified 25 studies—spanning 10 countries—that were included in the systematic review. CHW models were implemented alongside a variety of interventions, including behaviour change communication, supplementary foods, nutrition counselling, and integrated community health programmes. Key barriers in implementing successful CHW models include disruption of programmes due to active conflict, states of emergency, militancy, or political unrest; weak links between the community-based interventions and public health system; weak health system capacity that impeded referral and follow-ups; and cost of care and care-seeking. Key facilitators include CHWs’ connection to the community, close proximity of programmes to the community, supervision, and investment in high quality training and tools.

**Conclusions:**

The findings suggest that CHW models may be effective, cost-effective, acceptable, feasible, and scalable in the prevention, identification, and management child undernutrition in CAFS. The study findings also confirmed a need for greater evidence in the field. These findings may inform policymaking, programme implementation, and design to strengthen best practices for CHW models addressing child undernutrition in CAFS.

## Background

Malnutrition, specifically undernutrition, is a significant global challenge that contributes to nearly half of deaths in children under the age of five [1]. Malnutrition is described as *‘*deficiencies, excesses, or imbalances in a person’s intake of energy and/or nutrients*’* [1] and includes the condition undernutrition. Undernutrition is subdivided into four types: acute malnutrition,^1^ stunting,^2^ underweight,^3^ and micronutrient deficiencies. Acute malnutrition, is characterised by wasting—defined as a low mid-upper arm circumference (MUAC) and/or low weight-for-height z-score (WHZ)—and bilateral pitting nutritional oedema that often indicate a child has suffered recent and severe weight loss due to lacking sufficient food or having a disease that causes weight loss [2]. In 2022, approximately 148.1 million children under five were estimated to be suffering from stunting—defined as low height-for-age z-score (HAZ) and indicating chronic or recurring undernutrition [3]—and 45 million children were estimated to be suffering from wasting [3]. These conditions are often linked to a child experiencing adverse socioeconomic conditions, poor maternal health and nutrition, persistent illness, and/or improper infant and young child feeding (IYCF) and care.

The burden of undernutrition in children under five is disproportionately borne by conflict-affected, fragile, and low and middle-income countries (LMICs) [4, 5]. Conflict and violence cripple healthcare, economic, agricultural, labour, and supply chain systems, which in turn exacerbate food insecurity, hunger, and malnutrition [5]. The destruction and deterioration of housing and water, sanitation, and hygiene (WASH) infrastructure that are often associated with conflict and violence may cause mass displacement and overcrowding, leading to increased spread and risk of infectious diseases that in turn increase the risk of undernutrition [6]. Evidence indicates that conflict is the single greatest driver of hunger, with 60% of individuals suffering from hunger residing in areas experiencing war and violent conflict [7]. Children living in a conflict zone are more than twice as likely to suffer from malnourishment and four out of every five children whose growth has been stunted due to malnutrition today live in countries affected by conflict [8].

While food insecurity is a major cause of malnutrition, particularly in LMICs, food assistance has been reported as a short-term, unsustainable solution [9]. Instead, programmes that are integrated, intersectoral, sustainable, and community-based have been encouraged [10, 11]. Systematic reviews that investigated the efficacy of interventions in child malnutrition found that effective, evidence-based approaches to child stunting in LMICs were characterised by political commitment, multi-sectoral collaboration, community engagement, and community-based service delivery platforms [12].

Community health worker models have been employed in conflict-affected and fragile settings (CAFS) as an effective tool to expand and improve healthcare coverage, identify and treat illnesses, and ultimately, save lives and enable people to thrive [13]. The term community health worker (CHW) has been defined and described using a variety of methods, but generally refers to community-based health workers, often lay people from a community, that work within their community to monitor community health, assess community health needs, deliver health services, promote healthy behaviours, and receive training on different elements of community health and mobilisation [14]. CHW models are particularly useful in resource-constrained settings, extending weaker health systems so they can reach ‘last-mile’ communities and progress towards universal health coverage and health equity. These communities are difficult to access due to a multitude of reasons, including geographical isolation, distrust, poverty, marginalisation, and conflict, and disproportionality suffer from poorer health outcomes, including in child health and nutrition [13].

In places where access to health facilities is limited and disrupted—as is the case in low-resource and conflict-affected and fragile settings—CHWs and community-based interventions can be critical in detecting, referring, and managing cases of child malnutrition [15, 16]. The use of CHWs and community-based models in the identification, prevention, and management of child malnutrition in emergency settings has been advocated for by humanitarian actors and academic researchers alike [13, 15]. The United Nations, with a joint statement released by the World Health Organization (WHO), World Food Programme (WFP), and United Nations International Children’s Emergency Fund (UNICEF), emphasised the need to prioritise and integrate community-based management of SAM in emergency settings [17, 18].

There exist systematic reviews on delivering nutrition interventions to women and children in conflict settings [19] and the effectiveness of interventions in managing acute malnutrition in children under five in LMICs [20]. There is also a rapid review synthesis of lessons learned from community-based management of acute malnutrition programmes in CAFS [21]. However, to our knowledge, there lacks systematic evidence that comprehensively examines the impact of CHW models in preventing, identifying, and managing child undernutrition in CAFS. We conducted this review to explore the effectiveness, cost-effectiveness, ability, acceptability, and feasibility of CHW models in identifying, managing, and preventing undernutrition in children under the age of five in CAFS. These findings may inform policymaking, programme implementation, and programme design to strengthen best practices for CHW models addressing child undernutrition in CAFS.

## Methodology

### Study design

The review was conducted according to Preferred Reporting Items for Systematic Reviews and Meta-Analyses (PRISMA) reporting standards [22]. The search strategy was developed using the Population-Intervention-Comparisons-Outcomes-Setting (PICOS) framework [23] as a guide. We performed our search using Ovid online database search platform, specifically searching the databases of Ovid MEDLINE(R), COCHRANE, Embase Classic, Embase, Econlit, Global Health, SCOPUS, and Social Policy and Practice. Additional relevant articles were identified through reference-checking articles included from full text review stage onwards. Search terms were related to children being identified with or treated for malnutrition or undernutrition by CHWs in CAFS. The specific search terms and strategy used underwent multiple reviews, test searches, screenings, and iterations to ensure that the final search results retrieved as many relevant citations as possible. See Table 1 for final search terms and search strategy. The review was managed and conducted using Covidence review software [24]. No ethical approval was required for this systematic review; paperwork was submitted to the London School of Hygiene and Tropical Medicine MSc Research Ethics Committee to document the study and confirm that no ethical approval was needed.

**Table 1:** Final search terms and syntax for the Ovid online database search platform

#	Search strategy
1	community health worker* or village health worker* or village health team* or CHW* or VHT* or community health promoter* or community health aide* or community based* or community manage* or community health or CMAM or lay health worker* or lady health worker or volunteer health worker* or community health volunteer*
2	identif* or diagnos* or determin* or screen* or manag* or treat* or regulat* or handl* or administ* or address* or interven* or improv* or deliver*
3	child* or infant* or under five or paediatric
4	malnutrition or malnourish* or undernutrition or undernourish* or MAM or SAM or wasting or kwashiorkor
5	conflict* or fragile or war or combat or violen*
6	1 and 2 and 3 and 4 and 5

### Eligibility criteria

We aimed to gain a comprehensive understanding of effectiveness, cost-effectiveness, ability, acceptability, and feasibility of CHW models in the prevention, identification, and treatment of child undernutrition in CAFS. Therefore, we used a mixed-methods systematic literature review approach to identify and include quantitative, qualitative, and mixed-methods study designs. Peer-reviewed studies published between 2006 and October 2022 are included in our review. Grey literature, studies that were not peer-reviewed, and studies not published in English were excluded. Our rationale for the selected time frame of studies is twofold. First, we reasoned that the time frame needed to allow for focus on modern conflict-affected and fragile settings—when there has been a rise in active violent conflict that is more severe and protracted—and enable practical application of the systematic review to contemporary CAFS. Second, we required a standardisation method for conflict-affected and fragile settings. And, the selected time frame is when the World Bank Group’s (WBG) annual Fragile and Conflict-affected Situations (FCS) lists—which commenced publication in 2006 to classify study settings as non-CAFS or CAFS—became available.

The study population inclusion criteria were children under the age of five years old with or at risk of any type of undernutrition including, severe acute malnutrition (SAM), moderate acute malnutrition (MAM), global acute malnutrition (GAM), stunting, micronutrient deficiencies, and underweight. The intervention criteria were CHW models that were used to prevent, identify, or manage undernutrition, SAM, MAM, GAM, stunting, micronutrient deficiencies, and/or underweight in children under the age of five in CAFS. The outcome criteria included clinical outcomes (cure rate, defaulter rate, death rate, recovery rate, relapse rate), anthropometric outcomes (WAZ, HAZ, LAZ, WHZ, MUAC, bilateral pitting nutritional oedema), dietary diversity, minimum adequate diet, treatment coverage, IYCF indicators (including breastfeeding, exclusive breastfeeding, complementary feeding, and supplementary feeding), behaviour change, knowledge absorption, acceptability, feasibility, quality of care, costs, and cost-effectiveness. The settings inclusion criteria was community-based and CAFS—with CAFS defined using the WBG’s annual FCS list. The WBG classified fragile countries as such if they exhibited high levels of institutional and social fragility, which was determined through indicators measuring the quality of policy and institutions, and manifestations of fragility. Conflict-affected countries were determined by a threshold number of conflict-related deaths relative to the country’s population [25–29]. When applying the WBG FCS list to the inclusion and exclusion criteria for this systematic review, a study that had occurred in a country within a year of that country being classified as a fragile or conflict-affected situation was included in the literature as a CAFS. A study that had occurred in a setting that had been previously classified as a CAFS was included as a CAFS. See Table 2 for a full overview of the inclusion and exclusion criteria for this study.

**Table 2:** Study inclusion and exclusion criteria

Criteria	Inclusion criteria	Exclusion criteria
Population	Children under the age of five years old with or at risk of any of the following forms of malnutrition: severe acute malnutrition (SAM), moderate acute malnutrition (MAM), stunting, micronutrient deficiencies, undernutrition, and being underweight	Children over the age of five years or over 60 months old, and children without or not at risk of SAM, MAM, stunting, undernutrition, or being underweight
Geography	Conflict-affected and/or fragile settings (CAFS)	Non-CAFS
Time period	2006 until October 2022	Before 2006
Setting	Community-based setting, CAFS setting	Non community-based setting, non-CAFS setting
Intervention	Interventions identifying or managing risk or existence of SAM, MAM, stunting, micronutrient deficiencies, undernutrition, or underweight malnutrition in children under the age of five using a CHW model (this includes CHW models that work with community-based health facilities)	Interventions not targeting children with or at risk of SAM, MAM, stunting, micronutrient deficiencies, undernutrition, or being underweight. Interventions not involving the use of a CHW model
Outcome	Clinical outcomes (cure rate, defaulter rate, death rate, recovery rate, relapse rate, anthropometric outcomes (WAZ, HAZ, LAZ, WHZ, MUAC, bilateral pitting nutritional oedema), dietary diversity, minimum adequate diet, treatment coverage, IYCF indicators (including breastfeeding, exclusive breastfeeding, complementary feeding, and supplementary feeding), behaviour change, knowledge absorption, acceptability, feasibility, quality of care, and cost-effectiveness	Papers that did not report any of the outcomes of interest
Publication language	English	Non-English
Other characteristics	Peer-reviewed	Not peer-reviewed

### Data extraction and analysis

All retrieved papers were de-duplicated on the Ovid online search platform; unique records were imported into the Covidence platform. Any title lacking an abstract was hand-searched by title and author on Google, downloaded, and then imported into Covidence. To minimise variability between reviewers, the reviewers were supplied with a document detailing inclusion and exclusion criteria, as well as links to the WBG’s annual FCS lists from fiscal year 2006–2023. Two reviewers each conducted independent screenings on Covidence of all initial titles and abstracts to identify potentially relevant studies. Full text of relevant studies were obtained and loaded onto Covidence. Both reviewers then assessed the full text of each potentially relevant study for eligibility to include in the review. Discrepancies between reviewer decisions regarding title and abstract relevance and full text were flagged through Covidence and resolved through discussion.

As this was a systematic review of qualitative, quantitative, and mixed-methods study designs, methods for extraction and synthesis aimed to capture quantitative and qualitative data points. Information were extracted on country, study population, sample size and determination (if applicable), methods, intervention, comparator (if applicable), outcomes, findings, and facilitators or barriers to CHW effectiveness. A meta-analysis was not deemed to be appropriate for this study as the quantitative studies were few and not sufficiently homogenous. Instead, a narrative synthesis of the data was undertaken.

To appropriately assess the quality of each study according to their specific methodological design, the Critical Appraisal Skills Programme (CASP) tools were chosen. CASP checklists were used to assess the quality of qualitative studies, cohort studies, systematic reviews, randomised control trials, and economic evaluations [30]. One of the co-authors reviewed, assessed, and graded each study that had been included in the data extraction step, coding each study as high, moderate, low, or very low quality. No studies were excluded based on quality grade.

### Limitations

To the author’s knowledge, this systematic review is the first to focus specifically on CHW models being used to prevent, identify, or manage child undernutrition in CAFS. However, there are limitations to this systematic review and results shall be understood within the context of these limitations. The search results suggest limitations in the search strategy. Despite multiple test searches, reviews, and consultation with an academic librarian, the initial search string yielded a high volume of irrelevant studies, and the modified search string and syntax yielded a small number of better matched studies. We note that this limitation may be due to the exclusion of terminology, including stunting, underweight, and various terms related to micronutrient deficiencies, and hope the information provided here may inform future studies. We initially set the search time frame to be between 1992 and 2022; but the classification for CAFS were unsystematic and convoluted prior to 2006, when the WBG issued the FCS list. As such, we adjusted our time frame to capture studies between 2006 and 2022, ensuring that our results are not only comparable, but also reflect evidence on modern CAFS that policymakers and organisations can apply in current and emerging CAFS. This particular limitation may be due to the lack of a commonly used, standardised definition of CAFS. We used the WBG annual FCS lists that included only countries and the territory of the West Bank and Gaza to classify study settings. This type of country-level classification excludes countries and regions that border and are impacted by conflict; it also excludes conflicts such as those in the Kashmir region. Nevertheless, this is the most comprehensive list that we could identify and use to systematically classify different settings.

The diversity of study designs, outcomes investigated, and interventions researched led to significant heterogeneity, limiting the synthesis of results. We hope the inclusion of a variety of angles to holistically and effectively address child malnutrition in CAFs provided a range of modalities that may be useful in informing research, programmes, and policies. To ensure we included only results generated from robust methodology, we restricted the search to include only peer-reviewed publications. This decision inevitably excluded potentially relevant studies, particularly grey literature, conducted by humanitarian health and nutrition organisations. Programme reports from these organisations can be of particular importance as there is a dearth of published research in CAFS due to the challenges these settings present to research. Systematic reviews may also be prone to publication bias with interventions yielding null results being ignored; we caution against viewing the captured interventions as the only existing interventions. Instead, the included interventions shall be viewed as interventions with some evidence to suggest their ability, effectiveness, cost-effectiveness, acceptability, and feasibility.

## Results

### Search results

**Fig. 1:**
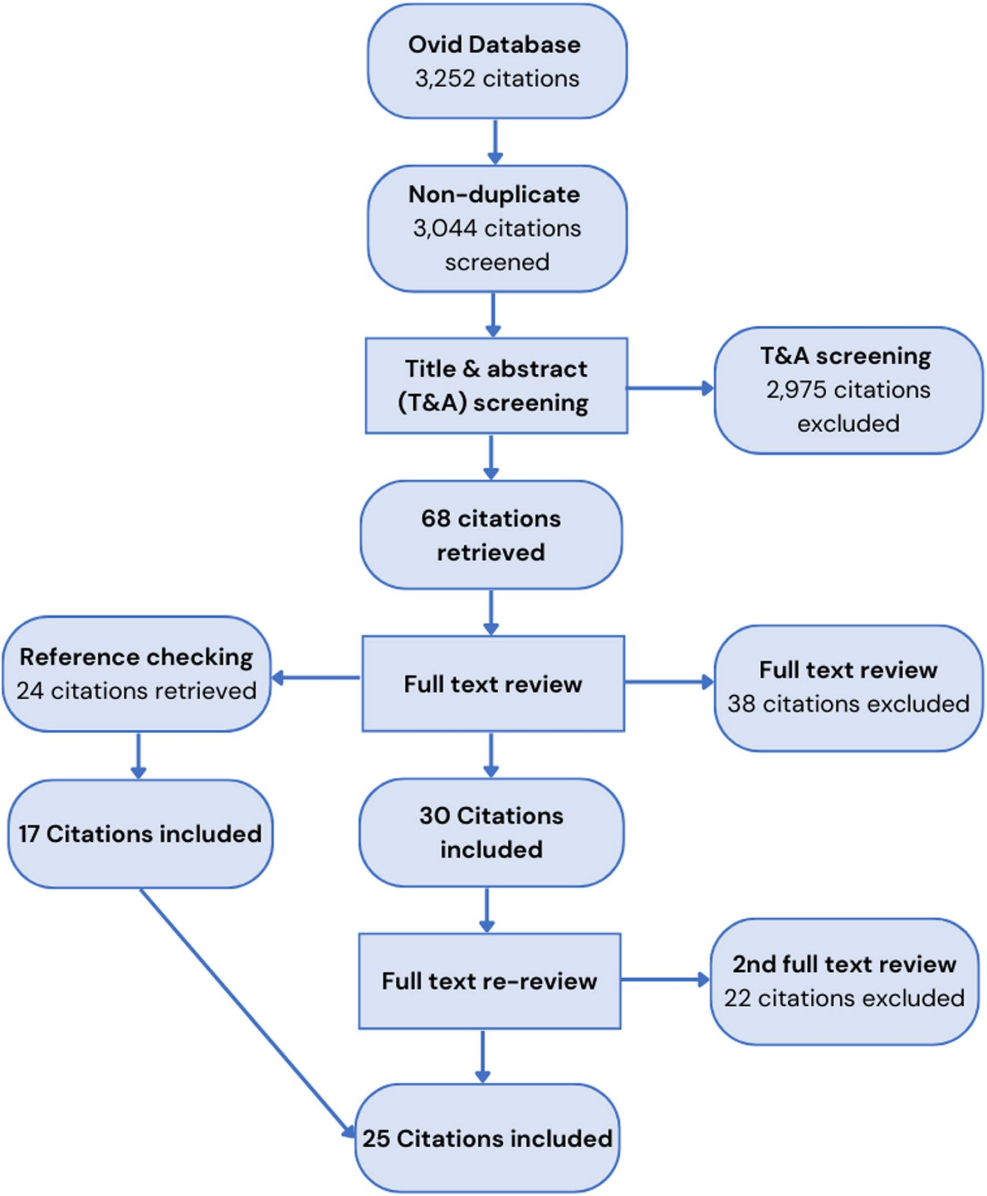
PRISMA diagram of included studies

The PRISMA diagram provided in Fig. 1 describes the search, screening, and review process for this study. A total of 3252 results were returned from the online academic database search platform Ovid. Upon import of these results into Covidence and deduplication, a total of 3044 citations remained for screening. An initial title and abstract screening conducted independently by two reviewers, resulted in 2975 citations being excluded. For the remaining 68 studies, a full text review was then undertaken, resulting in 38 further studies being excluded and 30 studies remaining for extraction. Of those excluded at the full text review stage, 16 studies were excluded due to being the wrong intervention, with many not being CHW interventions. Ten studies were excluded due to being the wrong study design, with many being grey literature. Eight of the studies were systematic, literature, or scoping reviews and, following the reference checking of these publications and identification of individual publications within each of them to include in this systematic review, they were excluded. An additional 24 citations were identified for full text review through reference-checking articles included in the full text review stage. Of those, 17 were ultimately included. Prior to extraction, the included publications were reviewed a second time to ensure they aligned with the updated inclusion criteria and WBG FCS lists. From this, an additional 22 studies were excluded, 10 of which were excluded because they were determined to not be in a CAFS. This process resulted in a final number of 25 studies meeting the inclusion criteria and being included in the review.

## Characteristics of included literature

We described the details of the included studies in this section. See *Appendix Table *3*: Summary table of study characteristics and findings* (ordered alphabetically) and *Appendix Table *4*: Summary table of study interventions* for summaries of the included studies.

### Study setting characteristics

The study settings of the 25 included studies spanned three regions and ten countries. The majority (84%) of publications included in the review researched interventions in the region of sub-Saharan Africa (SSA) and totalled 21 publications (n = 21). The next most common research setting region was Middle East North Africa (MENA), representing 12% of included studies and 3 publications (n = 3). Lastly, one publication’s research took place in Haiti in the Latin America region, representing 4% of included studies. Within sub-Saharan Africa, the subregion of West Africa had eight studies, the subregion of Central Africa had four studies, the subregion of East Africa had six studies, and the subregion of Southern Africa had three studies.

### Study design characteristics

The 25 studies eligible for inclusion were of quantitative, qualitative, or mixed-methods study designs. Five of the studies were randomised control trials (RCTs) investigating the effectiveness of various community-based malnutrition interventions involving a CHW model. Stewart et al. [31] conducted a multi-arm, cluster-RCT investigating the effectiveness of lipid-based nutrient (LNS) supplementation distributed by community nutrition workers (CNWs) on child anaemia and micronutrient status in Madagascar. Kurdi et al. [32] used data from a cluster-RCT on the Yemen Cash for Nutrition programme, which provided impoverished women that were pregnant or had children under two years old with monthly cash transfers conditional on their showing up to monthly community health volunteer (CHV)-led nutrition sensitisations. They used data on secondary outcomes of breastfeeding and water treatment to ascertain the impact of CHV-led nutritional training and counselling on IYCF practices and knowledge amongst participating women. Ayalew et al. [33] conducted an RCT studying the effect of complementary feeding behaviour change communication (BCC) through community-level actors in Ethiopia. A cluster-RCT by Maust et al. [34] measured the effectiveness of an integrated nutrition protocol—which included community-based, mother peer-counselling care groups with nutrition messaging delivered both on-site and at home visits—in managing SAM and MAM in Sierra Leone. A cluster-RCT by Lelijveld et al. [35] looked at the effectiveness of treating high-risk MAM with clinic-delivered therapeutic food and community elder delivered nutrition counselling versus just community elder delivered nutrition counselling in Sierra Leone.

Seven publications were cross-sectional comparison studies. Addo et al. [36] studied the combined effects of community-based nutrition education for mothers and pregnant women, community-based outreach counselling by CHWs on IYCF and small-quantity lipid-based nutrition supplementation (SQ-LNS), and the provision of SQ-LNS by CHWs on anaemia and growth in children in the Democratic Republic of the Congo (DRC). Locks et al. [37] studied the same programme, but instead looked at the impact of the integrated IYCF and SQ-LNS delivered by CHWs on outcomes of IYCF behaviour change and child dietary diversity in the DRC. Bisimwa et al. [38] researched the effectiveness of nutritional monitoring of children by community volunteers (CVs) in the DRC. Kim et al. [39] investigated the impact of community-based behaviour change interventions led by different cadres of CHWs on feeding practices and child stunting in Ethiopia. Mayhew et al. [40] evaluated the impact of a community-based growth monitoring and promotion (cGMP) programme using community health volunteers (CHVs) in Afghanistan on improving nutrition in underweight children. Worku et al. [41] examined the impact of child dietary diversity by the Sustainable Undernutrition Reduction programme in Ethiopia (SURE), a BCC project that used CHW cadres to enhance community-based nutrition (CBN) to address complementary feeding, improve household dietary diversity through IYCF, and sensitise on nutrition-specific agriculture. Getachew et al. [42] measured the accuracy of health extension workers (HEWs) in diagnosing child illnesses, including acute malnutrition.

Five of the study designs included in this systematic review were cohort studies. A retrospective, dual cohort study by Rajabi et al. [43] examined the relative effectiveness of supplementary feeding plus counselling by community respected elders compared with solely nutrition counselling by community respected elders in reducing risk of SAM and death in children with moderate wasting in Sierra Leone. A cohort study and impact evaluation were conducted by Balaluka et al. [44] in the DRC to determine the impact of CHWs promoting optimal breastfeeding practices through door-to-door visits and community meetings and organising monthly community child weighing sessions on exclusive breastfeeding (EBF) practices. An observational, clinical prospective multi-centre cohort study by Alvarez Morán et al. [45] researched the quality of care for uncomplicated SAM by CHWs in Mali. Conducted by the same primary author and in the same country was a multi-centre, randomised intervention study looking at the effectiveness of CHW models in identifying and treating SAM compared to a facility-based model [46]. Also in Mali, was a prospective, nonrandomised community intervention study by Charle-Cuéllar et al. [47] examining the effectiveness of different levels of supervision for CHWs when treating SAM.

Three of the publications included in the systematic review researched the acceptability and feasibility of interventions. Paul et al. [48] studied the impact of community-based complementary feeding messages on child feeding practices and used the Trials of Improved Practices (TIPs) methodology to understand the acceptability and feasibility of using local child nutrient supplements in Zimbabwe. Desai et al. [49] investigated the independent and combined effects of WASH practices and IYCF on child stunting and anaemia, the understanding of WASH and IYCF messages and tools piloted, and—using the TIPs methodology—the feasibility of delivering the intervention by village health workers (VHWs) in Zimbabwe. A study by Van Boetzelaer et al. [50] looked at the performance, feasibility, and community acceptability of community-based distributor (CBDs) with low literacy in treating SAM in South Sudan.

There were four studies that described using mixed-methods in their research, including the previously described Desai et al. [49] study, which conducted an effectiveness evaluation, TIPs acceptability and feasibility evaluation, and knowledge assessment of the VHW-delivered WASH and IYCF education interventions. Van Boetzelaer et al. [50] also used mixed methods, studying the performance of CBDs, child treatment outcomes, and community acceptability. Ayoya et al. [51] analysed both qualitative and quantitative data on community-based child nutrition interventions in Haiti. Renzaho et al. [52] used mixed methodologies to assess the impact and scalability of integrated, community-based programmes for the management of severe wasting in South Sudan.

Economic evaluations were conducted by two of the citations included in the systematic review, with both studies' research setting being Mali. Rogers et al. [53] evaluated the costs and cost-effectiveness of the treatment of uncomplicated SAM by CHWs in comparison to an outpatient facility in Mali. Isanaka et al. [54] conducted an incremental, cost-effectiveness analysis of community-based screening and treatment of SAM set within a cluster-RCT.

Lastly, one case study in Yemen by Tappis et al. [55] investigated the delivery of reproductive, maternal, new-born, child, and adolescent health and nutrition (RMNCAH + N) services.

## Narrative synthesis of results

### Ability of CHW models in treating child SAM in CAFS

Three studies assessed the ability and performance of CHWs in the treatment of SAM in CAFS. A cohort study by Alvarez Moran et al. [45] in Mali examined CHWs’ performance and ability to correctly treat uncomplicated SAM in children under five years old, with uncomplicated SAM defined by national protocol as MUAC < 11.5 cm, WHZ <  − 3, and/or nutritional oedema. The study reported that in 75% of cases CHWs correctly administered medical treatment for SAM, and in 100% of cases CHWs correctly administered RUTF for management of acute malnutrition or risk of acute malnutrition. Van Boetzelaer et al. [50] found that through conducting performance checklists, CBDs with low literacy in South Sudan were highly accurate in their adherence to a simplified SAM treatment protocol for uncomplicated SAM. Accuracy was determined through case management observations of CBDs by supervisory staff who then recorded a performance score. The performance score was made up of specific tasks for SAM treatment, including welcoming the caregiver, assessing for danger signs including bilateral pitting oedema, taking the child's MUAC measurement, conducting an appetite test, determining the weekly RUTF dosage based on the child's weight, administering medication (Amoxicillin for week 1, Albendazole for week 2), filling out the patient register, counselling the caregiver, child progress monitoring, and child discharge. During the study, 141 performance assessments were conducted, resulting in a mean performance score for CBDs of 89.9% (95% CI 86.4–96.0%) [50]. Furthermore, an investigation by Charle-Cuéllar et al. [47] into the effectiveness and performance of CHWs in treating uncomplicated SAM, using different models of supervision: high supervision, light supervision, and no supervision, found that the proportion of children cured was 81.4% in the high supervision group, 86.2% in the light supervision group, and 66.9% in the no supervision (control) group.

### Acceptability and feasibility of CHW models in preventing, identifying, and treating child undernutrition in CAFS

Four studies examined the acceptability and feasibility of CHW models in preventing, identifying, and managing child malnutrition in CAFS. A qualitative acceptability and feasibility analysis by Paul et al. [48] found that the VHW-delivered nutrition intervention involving BCC for complementary feeding methods, use of locally available complementary foods, and use of LNS, was widely accepted by the intervention community, with mothers eagerly absorbing and spreading the new knowledge they had received from the VHWs. The results of the study suggest that community-based approaches to creating and disseminating context-specific feeding messages were feasible and effective for the study setting [48]. Desai et al. [49] came to a similar conclusion for their study on the feasibility of VHWs in effectively delivering EBF, IYCF, and WASH promotion: that it is feasible for VHWs to deliver context-specific IYCF messages. Evidence also suggested that using low-literate CBDs to treat uncomplicated SAM in South Sudan was acceptable and feasible and that a simplified treatment protocol and tools adapted for low literacy were key [50]. A cGMP programme in Afghanistan delivered by CHWs was accepted by communities due to comprehensive community consultation; intentional, culturally acceptable, and accessible programme design; and high levels of participation amongst targeted children early in the programme [40].

### Effectiveness of CHWs in identifying child undernutrition

Four publications evaluated the effectiveness of CHW models in identifying child undernutrition in CAFS. A cross-sectional cohort study evaluated CVs’ effectiveness in monitoring the growth of children under five and identifying those highly susceptible to acute malnutrition in South Kivu, DRC—a context experiencing endemic malnutrition and armed conflict [38]. The monthly community weighing sessions conducted by CVs resulted in high rates of coverage (calculated as percentage of children weighed per village), even during periods of conflict, with the median percentage of children weighed per village ranging between 80 and 90% for those aged 12–59 months old and between 80 and 100% for those less than 12 months old. The median percentage of children aged 12–59 months old per village ranked as highly susceptible to malnutrition by the CVs decreased from 4.2% in 2004 to 2.8% in 2005. Another cross-sectional study in Ethiopia evaluated the diagnostic accuracy of HEWs when diagnosing common childhood illnesses, including malnutrition, and found that the ability of HEWs to correctly diagnose malnutrition was particularly poor, with only four out of ten children with malnutrition being correctly identified [42]. HEWs diagnosed MAM, uncomplicated SAM, and complicated SAM according to the WHO growth standards, using WFH/L, oedema, MUAC, presence of medical complications, the ability to finish an RUTF for children older than six months and the existence of a breastfeeding issue for children six months or younger. Overall, diagnostic sensitivity and specificity for child acute malnutrition by HEWs was 39% and 99%, respectively [42]. A cohort study in Mali looked at CHWs’ performance and ability to correctly screen for child uncomplicated SAM (defined by national protocol as MUAC < 11.5 cm, WHZ <  − 3, and/or nutritional oedema) and that, when screening, CHWs correctly assessed 97.6% of children for existence of cough, diarrhoea, fever, and vomiting; 95.2% of children for presence of major danger signs; 96.8% of children’s MUAC; 78.4% of children’s oedema; and 100% of children’s height [45]. Additionally, 77.8% of children who required an appetite test were tested correctly. A mixed methods study on the precis of nutrition in children in Haiti noted that CHWs were a key link to the health system, playing a central role in screening and follow up for malnutrition and services delivered outside the community [51].

### Effectiveness of child undernutrition behaviour change communication (BCC) interventions involving CHWs

Nine of the included studies investigated the effectiveness of community-based, BCC interventions in addressing child undernutrition and improving child growth and nutrition outcomes. Addo et al. [36] evaluated the impact of an enhanced IYCF programme—which included improved IYCF training for CHWs and outreach counselling conducted by CHWs—on anaemia and the anthropometric status of children in Katanga, DRC. The study results revealed that the intervention was associated with a 11.0% (95% CI − 18.1, − 3.8; *P *< 0.01) adjusted relative reduction in anaemia prevalence. However, there was no intervention effect on anthropometry, iron deficiency, or vitamin A deficiency. A cross-sectional comparison study by Mayhew et al. [40] researched how a cGMP programme carried out by illiterate CHWs impacted the nutritional status of Afghan children and found that children that had participated in the programme had a statistically significant higher mean WFA z-score of − 0.9 (95% CI − 1.0,  − 0.8), compared to − 1.2 (95% CI − .3,  − 1.1) for those that had not participated. Kim et al. [39] investigated the effectiveness of an intensive, community-based BCC intervention delivered by CHWs and community leaders on complementary feeding practices and child stunting in Ethiopia and found that while minimum dietary diversity and acceptable diet did increase significantly in the intensive group, they remained low at the endline (24.9% and 18.2%, respectively). Additionally, in children 6–23.9 months old, the study observed significant differential declines in stunting prevalence, with a decrease from 36.35 to 22.8% in the intensive group. Another BCC programme leveraged HEWs to improve dietary diversity amongst children aged 6–23 months; Worku et al. [41] found that children living in districts covered by the programme were 2.5 times more likely to have sufficient dietary diversity than those living in uncovered districts. A cluster-RCT by Ayalew et al. [33] investigated the effect of a community-based, complementary feeding BCC delivered by CHWs on infant growth and morbidity in rural communities in Ethiopia and found that, compared to the control group, infants in the intervention group had significantly higher weight gain (Mean Difference (MD) 0.46 kg; 95% CI 0.36–0.56) and length gain (MD 0.96 cm; 95% CI 0.56–1.36) and reduced rates of infant stunting (by 7.5% points; Risk Ratio (RR) = 0.68; 95% CI 0.47–0.98) and underweight (by 8.2% points; RR = 0.55; 95% CI 0.35–0.87).

A mixed methods pilot study by Desai et al. in Zimbabwe researched the effectiveness of VHWs' promotion of IYCF messaging on mothers’ child feeding practices and subsequent changes in child dietary diversity. It reported that maternal knowledge about infant feeding increased after receiving the VHW modules on IYCF [49]. Another Zimbabwe-based study by Paul et al. [48] on VHW-disseminated feeding messages found that there was a significant increase in intakes of energy, protein, vitamin A, calcium, zinc, iron, and folate from complementary foods after counselling (*p *< 0.05).

An RCT of the Yemen Cash for Nutrition programme—a programme that provided cash incentives to impoverished women that were pregnant or had children under two years old to attend monthly nutrition sessions by community health volunteers (CHVs)—found that programme exposure increased the probability of breastfeeding initiation within the first hour after delivery by 15.6% points (*p *< 0.05), the probability of exclusive breastfeeding (EBF) during the first six months by 14.4% points, the probability of households treating water consumed by adults by 16.7% points (*p *< 0.01), and the probability of treating water consumed by children under the age of two by 10.3% points (*p *< 0.10) [32]. The effectiveness of CHWs in promoting EBF was also explored in an area of the DRC with endemic malnutrition [44]. The study found that the median duration of EBF from birth was six months in the intervention group compared to four months in the comparison (the comparison group had no CHWs or community-based programme) group (*p *< 0.001). The intervention area also had a higher proportion of infants receiving EBF at six months old (57.7%), versus 2.7% in the comparison area (*p *< 0.001) [44].

### Effectiveness of CHW models in relation to supplementary feeding

Five of the included studies investigated the effectiveness of interventions combining CHW nutrition sensitisation and supplementary feeding in addressing child undernutrition and improving child growth and nutrition outcomes. A study by Rajabi et al. in Sierra Leone investigated the effectiveness of facility-based supplementary feeding plus nutrition counselling by community respected elders compared with solely nutrition counselling by community respected elders in reducing the risk of SAM and death in children with moderate wasting. It found that children in the supplementary feeding plus nutrition counselling cohort had a lower risk of developing SAM or dying over the 24-week period of follow up than children in the solely nutrition counselling cohort. The supplementary feeding cohort also had greater rates of weight gain and greater rates of MUAC gain when compared to the solely counselling cohort [43]. A cluster-RCT investigated the effectiveness of two treatments for high-risk MAM, with high-risk MAM defined as a child having one or more of the following criteria: MUAC < 11.9 cm, and/or WAZ <  − 3.5, and/or the mother not being the primary caregiver, and/or child being less than two years old [35]. The intervention consisted of ready-to-use therapeutic food (RUTF) provision, plus antibiotics, plus nutrition counselling; the control consisted of solely nutrition counselling [35]. The nutrition counselling for both arms was led by trained community elders who sensitised on IYCF and gave cooking demonstrations. The study results reported that children in the intervention arm had greater short-term recovery (48% compared to 39% at week 12; risk difference (RD): 0.08; 95% CI 0.03, 0.13), weight gain, and MUAC gain compared to the control arm. They also had lower risk of developing SAM (18% compared to 24%; RD − 0.07; 95% CI − 0.11, − 0.04) and dying (1.8% compared to 3.1%; RD − 0.02; 95% CI − 0.03, − 0.00) compared to the control arm. However, it was found that by week 24 the risk of SAM was similar in both groups [35].

The effectiveness of community-based supplementary feeding in conjunction with intensive community-based nutrition counselling, compared to just community-based nutrition counselling was further explored in a five-armed, cluster-RCT by Stewart et al. [31] in Madagascar. The control arm (T0), consisted of status quo treatment based on standard Madagascan growth monitoring and nutrition education protocol, this included monthly growth monitoring and key messages on maternal nutrition, early initiation of breastfeeding, EBF for the first six months, continued breastfeeding through two years old, and age-appropriate complementary feeding and hygiene behaviours. Community nutrition worker (CNWs) demonstrated cooking with local ingredients that were complementary foods. The government also distributed vitamin A biannually for children under 5 years old and pregnant women were given iron–folic acid supplements during ANC visits. There were four intervention arms: T1 consisted of T0 plus home visits for intensive nutrition counselling (defined as intensive due to additional CNWs being deployed for home visits); T2 consisted of T1 plus LNS for children aged 6–18 months that was distributed by CNWs; T3 consisted of T2 plus LNS for pregnant and lactating women; T4 consisted of T1 plus early childhood stimulation and parenting messages distributed by CNWs. It discovered that children in the study arms providing LNS had an approximately 40% lower prevalence of anaemia, as well as an approximately 25% lower prevalence of iron deficiency than children in the control study arm [31]. Locks et al. analysed the impact of an enhanced IYCF programme compared to the standard IYCF programme on breastfeeding, handwashing, and micronutrient deficiencies measured through child dietary diversity in DRC. The intervention was an enhanced IYCF programme consisting of both community‐ and facility‐based counselling for mothers on handwashing, SQ‐LNS, and IYCF practices; monthly SQ‐LNS distributions for children 6–12 months; and additional investments in the CHW platform including bike provision, enhanced training, updated CHW guidebook with images on IYCF, logbooks, improved/standardised supervision of CHWs, and standardised roles, responsibilities, and expectations for CHWs. The control was the national IYCF programme, with only facility-based counselling and no additional investments in the CHW platform [37]. The study found that the intervention group was associated with improvements in breastfeeding and handwashing behaviours, but not with significant change in dietary diversity [37]. In both groups, the minimum dietary diversity and minimum acceptable diet remained below 10% [37]. A study by Maust et al. exploring the recovery and coverage rates of an integrated protocol using RUTF for AM (intervention) versus standard management for AM (control). The standard management included mother peer-counselling care groups with nutrition messaging delivered on-site only, no home visits, treatment of MAM with fortified blended flour, treatment of SAM with RUTF, and WFH as the admission and discharge tool to the treatment programme. The integrated management of AM included mother peer-counselling care groups with nutrition messaging delivered both on-site and at home visits, treatment of MAM with fortified blended flour, treatment of SAM with RUTF, and MUAC used for admission and discharge, with a MUAC < 12.5 cm defining malnutrition. The study reported GAM recovery of 83% in the integrated protocol and 79% in the standard protocol. It also generated preliminary results that care group participation was associated with a greater recovery rate [34]. Coverage, calculated from number of children who received treatment over number of children eligible for treatment, was 71% for the intervention group, versus 55% in the control group (*P *= 0.0005) [34].

### Effectiveness of CHW models versus facility-based models

When comparing the effectiveness of a CHW model to the standard facility-based model in improving the coverage and treatment of SAM, a study by Alvarez Moran et al. [46], 2018 found that the CHW-enhanced intervention group had a cure ratio of 94.2%, while the health facility-based control group had an 88.6% cure ratio, giving a risk ratio of 1.07 (95% CI 1.01, 1.12). Mortality ratios between the two arms were very similar and not statistically significant [46]. Defaulter ratios in the control group were double the intervention group, while the coverage rate in the intervention group (86.7%) was more than double the coverage rate in the control group (41.6%) (*p *< 0.0001) [46].

### Costs and cost-effectiveness of CHW models in identifying and managing child undernutrition in CAFS

Two studies assessed the costs and cost-effectiveness of CHW models in identifying and managing child malnutrition in CAFS. Isanaka et al. included community-based screening in their economic evaluation of a study on the health outcomes, costs, and cost-effectiveness of a ‘do nothing’ strategy, ‘Treat SAM only’ strategy, and four dietary supplement strategies for MAM for screening and managing AM. Community-based screening costs were found to be only 4.7% of total costs for the ‘Treat SAM only’ arm and 1.7% to 1.9% of total costs for the MAM treatment arms. Costs were tabulated from the healthcare provider perspective, of which community-based screening was one component. The breakdown of costs for bi-monthly community-based screening for MAM treatment and SAM treatment were as follows. For MAM treatment, community-based screening costed 1.89 USD per MAM child identified, including 1.46 USD for personnel (77% of total activity), 0.26 USD for infrastructure and logistical support (14% of total activity), 0.17 for management and administration (9% of totally activity). For SAM treatment only, community-based screening costed 14.51 USD per SAM child identified, including 11.19 USD for personnel, 2.00 USD for infrastructure and logistical support, and 1.32 USD for management and administration [54]. Another study by Rogers et al. [53] estimated the costs and cost-effectiveness of CHW-delivered treatment for SAM in comparison with outpatient facility-based treatment for SAM. In the intervention arm, CHWs conducted nutrition sensitisations, screened for SAM, referred complicated cases, and treated uncomplicated cases in communities. In the control arm, CHWs conducted nutrition sensitisations, screened for SAM, and referred all cases to the outpatient facility. The study findings were that both provider and beneficiary costs were higher in the intervention arm than in the control arm. On the provider side, this was due to the greater role CHWs played in service delivery. On the beneficiary side, this was due to greater enrolment. However, at the individual household level, individual households in the intervention arm spent considerably less time (2.15 h in comparison to 3.92 h) and money (0.60 USD in comparison to 1.70 USD) than in the control arm. In terms of cost-effectiveness, CHW-delivered care was found to be more cost-effective than outpatient, facility-based care for treating uncomplicated SAM. The average cost per child treated by CHWs was 244 USD versus 442 USD in the outpatient facility. The average cost per child recovered was 259 USD by CHWs and 501 USD in the outpatient facility.

### Key barriers to CHW models in identifying or managing child undernutrition

Four studies reported barriers such as the interruption or disruption of programmes due to active conflict, states of emergency, militancy, or political unrest [38, 39, 52, 55]. Bisimwa et al. [38] reported an interruption in health facility services due to active conflict in Lwiro District, DRC, which corresponded to a 10% decrease in median percentage of children weighed monthly at community weighing sessions. However, they also noted that mean percentage of children weighed monthly never went below 80% and attributed the high mean percentage to the CVs’ and village nutrition committees’ ability to conduct the community-based nutrition intervention (including a public awareness campaign on malnutrition and growth monitoring, and arranging monthly weighing sessions) without the health facility [38]. In Ethiopia, a ten month state of emergency interrupted the implementation of an intensive IYCF BCC intervention using nutrition sensitisation, community mobilisation, and mass media campaign activities delivered by HEWs, HDTLs, AEWs, religious leaders, and CBOs [39]. Renzaho et al. [52] indicated that community-based management of severe wasting (CMSW) programmes in South Sudan were weakened by active conflict. Tappis et al. [55] cited insecurity as a main barrier to the effective delivery of both facility- and community-based RMNCAH + N services, with CHVs only able to address emergency needs (including child malnutrition needs) when the conflict environment allowed for movement and outreach to communities.

Two articles indicated weak links between community-based interventions and the existing public health system and overall weak health system capacity impeded referrals, health facility follow-ups, and the ability to provide care [52, 55]. Renzaho et al. observed a dearth of human resources for health in the form of well-trained CHWs and facility-based staff that could independently and effectively run CMSW programmes in South Sudan. The study also noted that the limited integration of CMSW programmes into existing local health infrastructure and national health budgets impeded government ownership of the CMSW programmes [52]. Tappis et al. [55] described the weak public health system in Yemen, and specifically the Ministry of Public Health and Population’s limited capacity to oversee and support health directorates, restricted the impact and activities of community-based RMNCAH + N service delivery.

The costs of care-seeking also hindered programme success, particularly when a programme was not home-based or proximate; namely, the time and transport costs were found to be an challenge [40, 50, 55]. In studying the impact of cGMP programme on the nutrition status of underweight children of participant caretakers in Afghanistan, Mayhew et al. [40] found that in the control group (the non-participants’ caretaker group), 52% of non-participants cited an absence of a nearby cGMP programme as the reason for not attending a cGMP programme. Ahead of Van Boetzelaer et al. [50] conducting their 2017 study in South Sudan on the performance of low-literate CBDs’ ability to treat SAM, only 41% of SAM children in the intervention area were enrolled in one of the International Rescue Committee’s static CMAM programmes, with caregivers citing the main barriers to accessing care as distance to health facilities, inaccessibility of health facilities during rainy season, and high opportunity costs. Tappis et al. [55] found that beneficiaries struggled to access facility-based RMNCAH + N care in Yemen due to transportation fees, far distance to reach functioning facilities, and roadblocks and checkpoints. Each of these studies made clear that static malnutrition interventions had a higher potential for inaccessibility for beneficiaries, and made the case for the use of mobile, CHW models that meet beneficiaries where they are.

Lack of demand due to distrust, disbelief in the helpfulness of the programmes, and the politicisation of aid was reported to have caused challenges for CHWs when delivering services to communities, with Mayhew et al. finding that 36% of the non-participant group did not attend a cGMP programme because they believed the programme would not be helpful; Van Boetzelaer et al. reporting that that high food insecurity and demand for RUTF made community members suspicious when a child was deemed ineligible for the CBDs' SAM treatment; and Tappis et al. citing that politicisation of aid led to restrictions in aid flow and service delivery based on local and geopolitical power dynamics [40, 50, 55]. In their study on the impacts of an enhanced IYCF programme-which included community- and facility-based counselling, SQ-LNS, and additional investments in the CHW platform-on IYCF practices and dietary diversity, Lock et al. [37] observed that lack of access to and purchasing power for healthy, nutrient-dense foods in communities not only hampered community members’ ability to access the foods they were sensitised to feed their children, but may have led to CHWs underemphasising nutrient-dense foods in their counselling as they knew the foods were difficult to acquire.

Three publications described weather-related barriers—with the study on CVs’ effectiveness in community-based child growth monitoring by Bisimwa et al. [38] noting the rates of acute malnutrition varying seasonally, the study on CMSW programmes in South Sudan by Renzaho et al. [52] reporting that wasting prevalence fluctuated with agriculture seasonality (remaining above the 15% emergency threshold during lean season), and Van Boetzelaer et al. [50] citing the inaccessibility of static CMAM services and programmes during the rainy season as previously described in the paragraph on costs of care-seeking. Tappis et al. [55] reported that an ongoing cholera epidemic hindered the effectiveness of CHWs’ work delivering RMNCAH + N services, with human resources, funding, technical expertise, and logistics being prioritized of cholera over other services. Mayhew et al. [40] reported that CHWs delivering the cGMP programme to underweight children faced challenges including low-literacy levels, lack of job aids, lack of security, and cultural norms around women (including female CHWs and female caretakers) needing to be accompanied by men in public. When considering the scalability of CHW models to address child malnutrition in CAFS, Renzaho et al. [52] found that CMSW programme scalability was hindered by a resource constraints, weak context-specificity frameworks for the CMSW programmes, and weak community mobilisation due to a lack of involvement of community members in CMSW programme leadership, while Alvarez Moran et al. [45] 2017 found that when working to scale up CHW treatment of uncomplicated SAM in Mali, balancing an increased workload was a barrier.

### Key facilitators to CHW models in identifying or managing child undernutrition

Key facilitators to successfully implementing CHW models and community-based interventions for child undernutrition were described in 15 of the included publications. The factor cited most widely, by six of the included articles, was the CHWs’ connection to the community, with studies reporting how it enabled influence and engagement with the community and families, social mobilisation, community trust, context-specificity, and community ownership and knowledge sharing [32, 33, 38, 40, 48, 50]. While investigating the effect of complementary feeding BCC disseminated through community level actors on infant morbidity and growth, Ayalew et al. [33] found that CHWs’ connection to the community enabled them to influence change and feeding practices. In a study evaluating CVs’ effectiveness in child growth monitoring and awareness raising for acute malnutrition, Bisimwa et al. [38], found that CVs effectively mobilised the community around the seriousness of malnutrition. Kurdi et al. studied the impact of the Yemen Cash for Nutrition program, which provided conditional cash transfers to impoverished women with young children to incentivise attendance at CHV-led nutrition sensitisations, on knowledge and practices related to breastfeeding, complementary feeding, and handwashing. They reported that the trainings being provided by women from the community (CHVs) allowed for a trusting relationship to form between the CHVs and participants, and for the programme to be run without strict oversight [32]. Van Boetzelaer et al. [50], in studying the performance of low-literate CBDs treating SAM in South Sudan, reported that, in general, caregivers expressed trust in CBDs and their ability to provide SAM treatment. A study by Paul et al. researching the impact of VHW-delivered complementary feeding messages and complementary feeding resources on infant diets, found that IYCF messages from CHWs that addressed context-specific barriers, context-specific local foods, and mothers directly were key to improving underlying diet and behaviour change. It also found that community ownership and knowledge sharing was spurred by VHW engagement [48]. Mayhew et al. [40] studied the impact of the cGMP programme on the nutritional status of young, Afghan children, and found that the CHW-delivered programme was culturally acceptable in both design and implementation.

Five articles cited that the close proximity of programmes, in the form of family-friendly home visits and local community sessions, facilitated interventions through eliminating beneficiary time and transport costs and delivering services in a comfortable, familiar setting [38, 40, 41, 44, 53]. Bisimwa et al. [38] noted that CVs living in the same area as participants and conducting weighing and nutrition sensitisations in the community supported effective monitoring of child growth and awareness raising on malnutrition. A study by Worku et al. [41] researched the impact of an HEW-delivered BCC nutrition education programme on child dietary diversity, and reported that household visits from HEWs were significantly associated with dietary diversity (AOR = 2.0; 95% CI 1.25, 3.21). Balaluka et al. [44] cited the proximity of CVs to breastfeeding mothers as a significant advantage in their study on CVs’ ability to improve breastfeeding of children under six months old in DRC. A cost-effectiveness study by Rogers et al. [53] reported that CHWs treating uncomplicated SAM was cost-effective, and that there were lower beneficiary costs incurred in the CHW-delivered SAM arm due to lower transport costs and opportunity costs (in the form of travel time). Lastly, Mayhew et al. [40] reported that conducting the cGMP programme through home visits and in villages led to greater attendance by study participants, which was associated with improved nutrition of underweight children in the study.

Two studies investigating IYCF BCC education through local community actors in Ethiopia cited that having a programme element focused on women’s participation through cooking demonstrations or food preparation was associated with behaviour change and improved child growth and dietary diversity [33, 41]. Ayalew et al. [33] reported that CHWs training women in local complementary food preparation contributed to infant weight and length gain, while Worku et al. [41] reported that children of women participating in CHW-led food preparation programmes had a dietary diversity AOR of 1.9 (95% CI 1.19, 2.96).

Supervision was cited as a key success factor in three of the included publications [37, 45, 50]. Four studies reported that investment in high quality training and tools, including active-learning practices of songs, practical exercises, role playing, and pictorial tools for illiterate CHWs, was essential to the success of the CHW programming [37, 40, 45, 50]. Locks et al. [37], studying the impact of an enhanced IYCF programme (which consisted of community- and facility-based counselling on IYCF practices; monthly SQ-LNS distributions; and investments in the CHW platform through enhanced CHW training, updated CHW guidebook with IYCF images, and improved/standardised supervision) on IYCF behaviours and dietary diversity attributed training and appropriate supervision of CHWs to success in improving breastfeeding and handwashing behaviours. Assessing the quality of care for CHW treatment of uncomplicated SAM, Alvarez Moran et al. found that well-trained and supervised CHWs could effectively manage uncomplicated SAM. Van Boetzelaer et al. [50] reported that low-literate CBDs could adhere to a simplified treatment protocol for uncomplicated SAM, with success facilitated through frequent supervision and the use of low-literacy adapted tools and training including use of songs, practical exercises, and role playing. Mayhew et al. [40] cited that providing pictorial tools for illiterate CHWs contributed to their effectiveness in the cGMP programme.

Two studies reported the beneficial use of incentives, with Bisimwa et al. [38], noting local government agreeing to employ CVs when there was a paid activity and Locks et al. [37] noting the use of bikes as transportation support and incentive. Van Boetzelaer et al. [50] found that ensuring a manageable workload for CHWs through having SAM treatment provided on a fixed day per week was key to CHWs' ability to effectively manage uncomplicated SAM. When considering facilitators for the scalability of CHW programmes, Renzaho et al. [52], found that the following were essential: best partnership practices, standardisation and adoption of a national programme implementation strategy, a BCC component to programmes, and an overall collaborative, holistic, and multidisciplinary approach to child malnutrition.

## Discussion

The confluence of the Covid-19 pandemic, climate change, and the global rise in violent, protracted conflict has led to catastrophic levels of hunger, food insecurity, and malnutrition in numerous regions and countries, such as occupied Palestinian territory, Sudan, Ukraine, and Yemen. [56]. Of the hunger hotspots identified as the highest concern for October 2022 to January 2023 by WFP and the Food and Agriculture Organization (FAO), conflict has been a key driver or aggravating factor for all of them [56]. Communities are being pushed onto the brink of or into famine, as is currently the case in Afghanistan, Ethiopia, the occupied territory of Gaza, Nigeria, Somalia, South Sudan, Sudan, and Yemen [56–58].

In these complex settings, the need for essential healthcare services is immense, but so are the challenges to delivering care to affected populations. As such, researching, designing, and implementing policy solutions that are effective, responsive, and adapted to the specific contexts and characteristics of child undernutrition in CAFS is critical. CHW policies and programmes have been deployed in both emergency and non-emergency settings to provide essential healthcare services for a variety of diseases, extend primary healthcare, and further universal health coverage. In recent years, the use of CHW models to address child malnutrition in CAFS has become more widely accepted, with the WHO endorsing the use of CHW-led treatment models for AM where possible in its revised guidelines on AM management [59]. It is critical that the body of research in this area grows alongside its popularity in order to ensure strong, continued, and effective advocacy for and integration of CHW models in local, national, and global policy agendas for child undernutrition in CAFS.

This systematic review identified 25 publications that reported on the effectiveness, cost-effectiveness, ability, feasibility, and acceptability of CHW models in preventing, identifying, or managing child undernutrition in CAFS. Identified evidence suggests that CHWs were effective in screening for child acute malnutrition (both MAM and SAM) in CAFS. Notably, one study reported that CVs in DRC were able to continue child growth monitoring and acute malnutrition screening activities and achieve high screening coverage even when the health facilities were closed during times of active armed conflict [38]. This finding suggests community involvement and CHWs play a key role in the building resilient child acute malnutrition screening programming in CAFS, which was echoed in a systematic review on nutrition interventions for women and children in CAFS [20].

In terms of diagnostic accuracy, one study on HEWs’ diagnostic accuracy in Ethiopia, found a concerningly low sensitivity of 39% for child malnutrition. HEWs diagnosed MAM, uncomplicated SAM, and complicated SAM according to WHO growth standards, using WFH/L, oedema, MUAC, presence of medical complications, the ability to finish an RUTF for children older than six months, and the existence of breastfeeding issue for children younger for children aged six months or younger [42]. Another study in Mali found CHWs classified SAM correctly in 100% of cases, measured height correctly in 100% of cases, correctly assessed oedema in 78.4% of cases, and correctly performed an appetite test in 77.8% of cases [45]. The incongruent results could be due to differences in training methods, cultural context, or CHW attributes.

All of the included studies on BCC (n = 9) found that this type of intervention to be effective, to varying degrees and with varying population sizes, in improving outcomes such as child feeding practices, breastfeeding practices, EBF, child diet, child dietary diversity, and child anthropometric indicators. These findings are in line with the results of a recent systematic review on the effectiveness of nutrition social and behaviour change communication (NSBCC) interventions and the results of a recent publication on BCC’s effectiveness in improving EBF and child anthropometric outcomes in Ethiopia [60, 61].

Evidence on the effectiveness of CHW nutrition counselling interventions and CHW nutrition counselling plus supplementary food interventions suggest that a CHW intervention that only employs nutrition counselling is not effective in improving child growth, improving dietary diversity, or preventing the deterioration of acute malnutrition. However, CHW nutrition counselling can be impactful when used in conjunction with supplementary foods. No publications included in this systematic review researched whether the provision of supplementary foods is effective without the use of nutrition counselling and CHWs. In addition, little information was provided about the contents and implementation of the nutrition counselling in the included studies. These findings reflect similar results from a systematic review researching the use of food products to manage MAM and suggest a greater need to investigate the role of CHWs and nutrition counselling in the effectiveness of supplementary food interventions [62].

Both publications conducting economic evaluations on CHW models for the screening and management of uncomplicated SAM found the CHW interventions to be cost-effective. These results align with those of a recent systematic review on the costs and cost-effectiveness of child undernutrition treatment to households, health providers, organisations, and governments in LMICs [63]. Acceptability and feasibility were also found to be high in the three publications that looked at these indicators, with community engagement, social mobilisation, and cultural appropriateness playing important roles.

When synthesising publications’ findings on key barriers and facilitators to the CHW interventions, it became clear that while some elements were inherently barriers (such as insecurity, violence, and political unrest) or facilitators (such as community engagement), many of the key barriers or facilitators were not inherently challenging or supportive towards the success of the CHW model. Instead, they were key factors that, depending on the degree to which they were leveraged and integrated into programme design, became a challenge or a benefit. Some of these flexible factors include proximity of programming for beneficiary communities, degree of context specificity, level of community mobilisation, quality of training, and degree of integration of programmes.

Overall, the publications included in the review suggested that CHW models were effective, cost-effective, feasible, and acceptable in preventing, identifying, and managing child undernutrition. However, the success of the CHW model was reported to varying degrees, with some dependency on the type of intervention the CHWs delivered. In addition, the heterogeneity of the included publications with regards to study designs, outcomes, measurements, and interventions, while providing a comprehensive view of CHW child undernutrition work being done in CAFS, makes it difficult to synthesise across studies and generalise results, presenting significant challenges for policy application purposes. Future studies shall aim to harmonise data gathering across programs while still allowing for the capturing of context-specific information. Such aim may be accomplished through convening a working group, that includes agencies, researchers, CHWs, policymakers, and affected populations, to establish a set of global definitions, indicators, and elements to report on, as well as templates, protocols and guidelines to follow. This shall include discussion of the options for CAFS classification, and recommendations for how and when to apply different classification methodologies. In addition, the use of clear definitions and in-depth descriptions of interventions, models, and implementation methods would allow for the extraction of valuable data, without confusion around what type of programme is being studied and by whom it is being delivered.

This systematic review was intentionally broad in its inclusion of different CHW interventions (screening, BCC, supplementary foods, identification, management, etc.), study designs (qualitative, quantitative, and mixed-methods), types of undernutrition addressed (AM, SAM, MAM, wasting, stunting, underweight, and micronutrient deficiencies) and outcomes (effectiveness, cost-effectiveness, ability, feasibility, acceptability, and scalability). The rationale is to generate a comprehensive review that could be leveraged for holistic policy and programme design in the field. While the review covered these various elements of CHW interventions for child undernutrition in CAFS, it revealed that little information exists on the effectiveness, cost-effectiveness, ability, feasibility, acceptability, and scalability of CHW models in preventing, identifying or managing child undernutrition in CAFS.

The identified evidence suggests that CHW models have the potential to deliver effective, empowering, equitable care for child malnutrition, while fostering resilience amongst conflicted-affected populations. The effective use of CHWs models requires an integrated, multisectoral, community-centred, and context-appropriate policy approach to be considered and embedded in policy, programme design, and implementation. To ensure strong community acceptability and mobilisation, CHWs and community members shall be engaged as co-designers, programmes shall be designed to minimise the distance needed to travel to receive services (with home visits being particularly effective), and strong, supportive links shall be created for any referral services needed from other parts of the healthcare system. To maximise CHW performance and ability, specific attention shall be paid to supervision, incentivisation, and training of CHWs. To inform policy design and implementation, anthropological research of the specific community context and desk research of similar contexts where CHW programmes have been implemented shall be undertaken.

## Conclusion

This systematic review identified evidence to support the hypothesis that CHWs can play a key, beneficial role in the prevention, identification, and management of child undernutrition in fragile or conflict-affected settings, with CHW models being reported to be effective, cost-effective, acceptable, feasible, and scalable. The strength of CHW interventions rests on key factors including the degree of community engagement, service proximity, presence of supervision, and quality of training. However, the sparse literature and scarcity of evidence weaken the findings; this is an issue that requires investment and attention from the field in order to build informed, sustainable, context-specific policy solutions for addressing child undernutrition in CAFS.

## Data Availability

The datasets used and/or analyzed during the current study are available from the corresponding author on reasonable request.

## Appendix

See Table 3 and 4.

**Table 3:** Summary table of study characteristics and findings (ordered alphabetically)

Title, author, and year	Country CASP grade	Aims	Study population and sample size	Methods (key words)	Intervention and comparator	Outcomes	Key study results & findings	Type of malnutrition	Barriers to and facilitators of CHW effectiveness
**Severe and Moderate Acute Malnutrition Can Be Successfully Managed with an Integrated Protocol in Sierra Leone** Maust et al. [34]	Sierra Leone -—-—-—- **CASP** Low	Evaluate coverage & recovery rates for GAM of an integrated protocol with RUTF compared to a standard protocol	1957 children under 5 years old	Cluster-RCT	**Intervention** Integrated management of GAM included mother peer-counselling care groups with nutrition messaging delivered both on-site & at home visits; MUAC for admission and discharge, with a MUAC < 12.5 cm defining malnutrition **Comparator** Standard management included messaging on-site & no home visits; treated MAM with fortified blended flour and SAM with RUTF, with WFH as the admission tool to treatment programme	Coverage (calculated from number of children who received treatment over number of children eligible for treatment); recovery, remaining malnourished, death, or lost to follow up; *recovery not equivalent between two study arms because used different anthropometric measurements to determine malnutrition (MUAC in intervention, WHZ in control)	Majority of children in the intervention arm had MAM (774 of 1100; 70%) versus most children in the control arm having SAM (537 of 857; 63%; *P *= 0.0001). Coverage for the intervention group was 71%, versus 55% in the control group (*P *= 0.0005); GAM recovery was 910 of 1100 (83%) children in the intervention group and 682 of 857 (79%) children in the control group; care group participation was associated with higher recovery rates, suggesting that sensitising & emphasising good nutrition & hygiene practices may be important to integrate alongside feeding programmes for children with GAM. This observation is seen to be preliminary due to lack of data collection on care group attendance or experiences	GAM	
**Improving nutrition in Afghanistan through a community-based growth monitoring and promotion programme: A pre-post evaluation in five districts** Mayhew et al. [40]	Afghanistan -—-—-—- **CASP** Moderate	Determine impact of community-based growth monitoring and promotion (cGMP) programme on the nutritional status of young, Afghan children	828 caretakers & children; 414 children under 2 years old, 414 caretakers	Cross-sectional comparison of mean WAZ between participants and non-participants, matching assigned sex at birth, age, & geography; retrospective comparison between initial & final survey of WAZ in cohort of cGMP participants meeting evaluation’s inclusion criteria	**Intervention** cGMP where CHWs deliver regular growth-monitoring sessions for children under 5 years old and nutrition counselling for caretakers **Comparator** Non-participants selected based on demographic criteria matching and consent from caretaker for child to be included in the evaluation	WAZ; programme acceptability	Children participating in the cGMP programme had a statistically significant higher mean WAZ, − 0.9 (95% CI − 1.0, − 0.8), than those that didn’t participate, − 1.2 (95% CI − 1.3, − 1.1). For the intervention children, mean WAZ change was the same for both the last cGMP visit and evaluation visit, a statistically significant increase of 0.3 (95% CI 0.2, 0.5) WAZs. There was no association between nutritional outcomes and the literacy level of caretakers. The programme was accepted by communities. The cGMP programme in Afghanistan for illiterate women can help improve child nutrition, specifically in underweight children who enter the programme at under 9 months old and attend half of the sessions or more	Underweight	**Barriers to CHWs:** low literacy levels, lack of job aids/ tools to address malnutrition, insecurity, and cultural norms requiring women to be accompanied by a male relative when in public **Barriers to beneficiary community:** distance from programme site; don’t believe in programme **Facilitators:** extensive community consultation; culturally acceptable programme design and implementation; high levels of participation amongst targeted children early in the programme; children entering programme at under 9 months old; regular attendance (facilitated by occurring villages and homes); pictorial tools for illiterate CHWs
**Complementary feeding messages that target cultural barriers enhance both the use of lipid-based nutrient supplements and underlying feeding practices to improve infant diets in rural Zimbabwe** Paul et al. [48]	Zimbabwe -—-—-—- **CASP** Moderate	Evaluate feasibility of improving infant diets using (1) only locally available resources & (2) locally available resources plus 20 g of LiNS	32 children, two age groups (6–8 months and 9–12 moths); 8 per age group per round	Qualitative acceptability & feasibility study of BCC; conducted 2 rounds of 2-week home interventions: 1st round to discern how infant diets in rural Zimbabwe could be improved without introducing a novel commodity; 2nd round to introduce LiNS, Nutributter® & also improve diet quality with local foods	**Intervention** Supplementation with lipid-based nutrient supplements (LiNS), complementary feeding messaging, & counselling from VHWs **Comparator** Pre-intervention (baseline indicators)	Whether consumption of key complementary foods increased after counselling; intakes of energy, protein, vitamin A, folate, calcium, iron and zinc from complementary foods; acceptability of the programme from the community	Energy, protein, vitamin A, folate, calcium, iron and zinc intake from complementary foods increased a significant amount after counselling & wasn’t dependent on being given Nutributter (*P *< 0.05). Intakes of fat, folate, iron, and zinc increased solely (fat) or at an increased amount (folate, iron, and zinc) when given Nutributter (*P *< 0.05). While providing LiNS was essential to making sure sufficient intakes of iron and zinc, educational messages that addressed context-specific barriers & addressed mothers directly were key to improving underlying diet and behaviour change; Nutributter was acceptable to mothers and children	Micronutrient deficiencies	**Facilitators:** context specificity of VHWs working with mothers; community ownership/knowledge sharing spurred by VHW engagement; infant feeding messages context-specific about local foods
**Nutritional training in a humanitarian context: Evidence from a cluster randomized trial** Kurdi et al. [32]	Yemen -—-—-—- **CASP** Low	Assess the Yemen Cash for Nutrition programme’s impact on the knowledge and practices related to breastfeeding & water treatment for impoverished women that are pregnant or have young children	1945 impoverished women either pregnant or with children under 2 years old in 190 clusters randomly assigned to treatment versus control	Data from cluster-RCT; primary outcome: child HFA; secondary immediate outcomes IYCF knowledge & behaviour change; panel household questionnaire to collect data	**Intervention** The Cash for Nutrition programme was a pilot conditional cash transfer programme with the objective of reducing the high prevalence of child malnutrition, through targeting impoverished women who had children under 2 years or were pregnant at the time of enrolment. Participants received monthly cash transfers conditional on showing up to monthly, CHV-led nutrition sensitisations. Each CHV had a catchment area of several villages and were recruited to become CHVs for the programme from among women living in the targeted area between 18 and 35 years and educated up to secondary school. CHVs conducted quarterly screening sessions using MUAC to detect and refer malnutrition cases	Impact of programme on self-reported practices of early initiation of breastfeeding, EBF, water treatment, & complementary feeding; knowledge of topics covered in nutritional training sessions	Similar impacts on knowledge and breastfeeding between literate and illiterate women; community nutritional training sessions in a cash transfer humanitarian response model can be effective at changing behaviour; programme increased probability of breastfeeding initiation within the first hour after delivery by 15.6% points (*p *< .05; control = 74.4% and treatment = 83.6%), the probability of exclusive breastfeeding during the first 6 months by 14.4% points (control = 13.5% and treatment = 25.3%), the probability of households treating water consumed by adults by 16.7% points (*p *< .01; control = 13.9% and treatment = 23.4%), and treating water consumed by children under two by 10.3% points (*p *< .10; control = 31.2% and treatment = 37.9%)	Not specified	**Facilitators:** trainings provided by women from local community, allowed trusting relationship between the participants and CHVs and for programme to be run without strict oversight
**Treating high-risk moderate acute malnutrition using therapeutic food compared with nutrition counseling (Hi-MAM Study): a cluster-randomized controlled trial** Lelijveld et al. [35]	Sierra Leone -—-—-—- **CASP** Low	Discern if giving RUTF and antibiotics alongside nutritional counselling to children at “high-risk” of MAM (HR-MAM) would lead to better recovery and less deterioration than solely nutrition counselling	Children aged 6–59 months 22 cluster sites; Intervention; 573 children Control: 714 children Sample size: around 800 with MAM across 20 cluster sites	Cluster-RCT; outcomes compared with intention-to-treat analysis	**Intervention** Children classified as high-risk MAM (HR-MAM) or low-risk MAM (LR-MAM). HR-MAM group given 1 daily packet of RUTF (until MUAC > 12.4 cm achieved) & amoxicillin; children attended clinic every other week until treatment complete & returned for follow-up at 12 & 24 weeks post-enrolment. Caretakers participated in mother support groups delivered by a community respected elder twice a week, with 4 nutrition sessions on optimising IYCF, cooking, WASH, health care seeking, child development, & MUAC for mothers **Comparator** 6 weeks of nutrition counselling only in the form of mother support groups where community respected elders delivered sessions to caretakers, every other week, 4 nutrition sessions about optimizing IYCF, cooking demonstrations, WASH, health care seeking, child development, & training on MUAC for mothers	HR-MAM defined as having ≥ 1 of the following criteria: MUAC < 11.9 cm, WAZ < − 3.5, mother not primary caregiver, or child < 2 years not breastfed; recovery rate; risk of SAM; risk of death; MUAC; WAZ	**Intervention:** 317 (55%) classified as HR-MAM; greater short-term recovery at intervention sites; children had lower risk of progressing to SAM (18% intervention compared with 24% control; RD: − 0.07; 95% CI − 0.11, − 0.04), lower risk of death (1.8% intervention compared with 3.1% control; RD: − 0.02; 95% CI − 0.03, − 0.00), and greater increases in MUAC and weight than control children. However, by 24 weeks, the risk of SAM between the intervention and control groups were similar	MAM (HR-MAM and LR-MAM); SAM	
**An integrated infant and young child feeding and small‐quantity lipid‐based nutrient supplementation programme in the Democratic Republic of Congo is associated with improvements in breastfeeding and handwashing behaviours but not dietary diversity** Locks et al. [37]	DRC -—-—-—- **CASP** Low	Analyse impact of enhanced IYCF programme, an integrated IYCF—SQ-LNS programme, on IYCF and hand washing practices	Children aged 6–18 months **Baseline:** N-intervention = 650; N-control = 638; **Endline:** N-intervention = 654; N-control = 653	Cross‐sectional preintervention and postintervention surveys conducted; difference in differences (DiD) analyses used mixed linear regression models	**Intervention** Enhanced IYCF programme: community‐ and facility‐based counselling for mothers on handwashing, SQ‐LNS, and IYCF practices; monthly SQ‐LNS distributions for children 6–12 months; additional investments in CHW platform including bike provision, enhanced training, updated CHW guidebook with images on IYCF; logbooks; standardised roles and responsibilities expectations; & improved/standardised supervision **Comparator** National IYCF programme: facility‐based IYCF counselling with no SQ‐LNS distributions, no investments in MM & IEC, no additional investments in CHW platform	Breastfeeding practices in the first 6 months; handwashing practices during food & defecation in previous day; dietary diversity (proportion of children fed minimum dietary diversity or minimum acceptable diet)	Greater increases in proportion of intervention mothers compared to control mothers recalling: initiating breastfeeding within 1 h of birth (Adjusted DiD [95% CI]: + 56.4% [49.3, 63.4], *P *< 0.001), waiting until 6 months to give water (+ 66.9% [60.6, 73.2], *P *< 0.001) and complementary foods (+ 56.4% [49.3, 63.4], *P *< 0.001), meeting minimum meal frequency the day prior (+ 9.2% [2.7, 15.7], *P *= 0.005); knowledge about anaemia (+ 16.9% [10.4, 23.3], *P *< 0.001); having soap (+ 14.9% [8.3,21.5], *P *< 0.001); & washing hands after going to the bathroom, before preparing food, & before child feeding the day prior (+ 10.5% [5.8, 15.2], + 12.5% [9.3, 15.6] and + 15.0% [11.2, 18.8], respectively, *p *< 0.001 for all). Enhanced IYCF associated with positive changes in IYCF practices, but not dietary diversity (minimum dietary diversity and minimum acceptable diet were similar and below 10% for both groups); intervention mothers had a high likelihood of recalling getting IYCF messages from CHWs; in the intervention site, mothers with high programme (and CHW) exposure were more likely to wait until 6 months to introduce water or complementary foods versus mothers with low programme exposure	Micronutrient deficiencies	**Barriers:** lack of access or purchasing power to acquire nutrient dense/dietarily diverse foods; health workers may not emphasize messages because they know the foods are difficult to get **Facilitators:** training; appropriate supervision in IYCF; bike as transport support and incentive
**Cost-effectiveness of community-based screening and treatment of moderate acute malnutrition in Mali** Isanaka et al. [54]	Mali -—-—-—- **CASP** High	Approximate health outcomes, costs, & cost-effectiveness 4 dietary supplements used to treat MAM in children 6–35 months old	95 study villages; 12 community health centres	Incremental cost-effectiveness analysis within a cluster-RCT; costs estimated from the healthcare provider perspective; categorising & costing was done for all resources for bimonthly community-based screenings & MAM treatment; estimations were done with an ingredients approach	**Intervention** CVs conducted community-based screening for MAM and SAM using MUAC every 2 months 12 community health centres randomised to deliver one of 4 dietary supplements: **RUSF:** ready-to-use, enriched soy protein, peanut paste **CSB + + :** corn–soy blend with soybean flour, maize flour, dried skimmed milk, soy oil & micronutrients **Misola (MI):** locally produced, micronutrient-fortified, cereal-legume blend with millet or maize, soy, & peanut flour **LMF:** locally milled flour mixture, including millet, beans, oil & sugar **Comparator** No dietary supplement	Cost per MAM or SAM child identified	**Key findings:** Community-based screening has the potential to promote early case detection and increase treatment referrals and makes up a relatively small sum in the management of AM **Costs for bi-monthly community-based screening:** For MAM treatment: 1.89 USD per MAM child identified, including 1.46 USD for personnel (77% of total activity), 0.26 USD for infrastructure and logistical support (14% of total activity), 0.17 for management and administration (9% of totally activity); constituted 1.7–1.9% of costs for MAM treatment arms For SAM treatment only: 14.51 per SAM child identified, including 11.19 USD for personnel, 2.00 USD for infrastructure and logistical support, and 1.32 USD for management and administration; constituted 4.7% of costs of ‘Treat SAM only’ arm	Acute malnutrition (MAM and SAM)	Cost-effective
**Behavior Change Interventions Delivered through Interpersonal Communication, Agricultural Activities, Community Mobilization, and Mass Media Increase Complementary Feeding Practices and Reduce Child Stunting in Ethiopia** Kim et al. [39]	Ethiopia -—-—-—- **CASP** Moderate	Assess the impact of an intensive BCC intervention compared with standard interventions on child feeding practices, caregiver knowledge, & anthropometric outcomes	Sample size of 2700 (1350/group) children; baseline total (n = 2646); endline total (n = 2720) Children aged 6–23.9 months	Cluster-randomised, nonblinded impact evaluation design with cross-sectional surveys	**Intervention** Intensive BCC interventions: HEWs and health development team leaders (HDTLs) sensitised through interpersonal communication activities (IPC) on IYCF during health post visits and home visits and food demonstrations; AEWs promoted nutrition-sensitive agricultural activities (AG) that help with agriculture and child growth; religious leaders delivered IYCF-focused community mobilisation (CM) activities about adequate child feeding during fasting, & CBOs facilitated enhanced community conversations about IYCF. There was also a mass media (MM) campaign on IYCF practices **COMPARATOR** Nonintensive areas: HEWs, HDTLs, and AEWs provided the regular services; few, if any IYCF CM activities; no directed MM	**Primary outcome:** WHO core CF practices: minimum dietary diversity; minimum meal frequency; minimum acceptable diet; consumption of iron-rich or iron-fortified foods; timely introduction of solid, semisolid, or soft foods. Based on maternal 24-h recall of foods consumed **Secondary outcomes:** maternal knowledge about CF and stunting prevalence among children; assessed based on mothers’ responses to a set of 12 questions about CF; anthropometric data: HAZ, WAZ, and WHZ	**Intensive group endline:** IPC exposure was 17.8–32.3%, AG exposure was 22.7–36.0%, CM exposure was 18.6–54.3%, MM exposure was 35.4%; minimum dietary diversity and minimum acceptable diet increased significantly but remained low at endline (24.9% and 18.2%, respectively). There were significant differential declines in stunting prevalence (DDE: − 5.6 percentage points; *P *< 0.05) in children 6–23.9 months old, decreasing from 36.3% to 22.8% in the intervention group. Dose–response analyses showed higher odds of minimum dietary diversity (OR: 3.3; 95% CI 2.2, 4.8) and minimum meal frequency (OR: 1.9; 95% CI 1.4, 2.6) and higher HAZ (β: 0.24; 95% CI 0.04, 0.4) among women exposed to 3 or 4 of the IYCF BCC platforms. Path analyses revealed a strong relation between AG and egg consumption, which led to increased HAZ and child dietary diversity		**Barriers:** Disruptions in programme implementation due to state of emergency for 10 months
**Nutritional Monitoring of Preschool-Age Children by Community Volunteers during Armed Conflict in the Democratic Republic of the Congo** Bisimwa et al. [38]	DRC -—-—-—- **CASP** Low	Evaluate CVs' effectiveness in child growth monitoring in an area of endemic malnutrition & armed conflict in South Kivu	5479 children aged under 5 years old	Cross-sectional comparison; effectiveness evaluation	A community-based nutrition programme in the Lwiro Health sector, lasting for 32 months, and including a public awareness campaign, recruitment & training of CVs, & arrangement of monthly community weighing sessions	WFA, oedema, median percentage of children weighed per village for children of 12–59 months old; percentage of children weighed per village for children under 12 months; median percentage of children 12–59 months old per village ranked as highly susceptible to malnutrition by CVs	**Key findings:** CVs can be an important support to the health system through effectively decentralizing nutritional monitoring of pre-school aged children at the community level **Median percentage of children 12–59 months old weighed per village:** between 80 and 90% **Median percentage of children < 12 months old weighed per village:** between 80 and 100% **Median percentage of children 12–59 months old per village ranked as highly susceptible to malnutrition:** decreased from 4.2% (range, 0% to 35.3%) in 2004 to 2.8% (range, 0.0% to 18.9%) in 2005	Endemic malnutrition, acute malnutrition	**Barriers:** malnutrition rates varied seasonally; interruption of health facility services by active conflict **Facilitators:** sessions proximate to home (CVs lived in same area); weighing sessions in friendly family context, effective social mobilization; incentives: motorbikes, DHO agreed to employ CVs whenever there was a paid activity; support from community leaders; community knowledge & involvement in child growth monitoring
Scaling severe acute malnutrition treatment with community health workers: a geospatial coverage analysis in rural Mali Charle-Cuéllar et al. [47]	Mali -—-—-—- **CASP** Moderate	Investigate the most effective supervision model for providing SAM treatment through CHWs	6112 children aged 6–59 months	Prospective non-randomized community intervention trial; three arms: two intervention arms & one control arm, distinguished by different levels of supervision	**Intervention** **High supervision arm:** supportive supervision for iCCM as well as nutrition-specific supervision **Light supervision arm:** supportive supervision based on iCCM package **Comparator** No specific supervision	**Primary outcome:** cure rate, WHZ ≥ −1.5 or MUAC ≥ 125 mm & absence of nutritional oedema for two consecutive visits **Secondary outcomes:** defaulters, deaths, referrals with complications, quality of care delivered by CHWs	Proportion of children cured: 81.4% in the high supervision group, 86.2% in the light supervision group, & 66.9% in the control group. Children treated by CHWs with some form of supervision had better outcomes than those treated by CHWs with no supervision (*p *< 0.001). There was no significant difference between light & high supervision groups. CHWs with high supervision did perform better in the majority of tasks assessed	SAM	
**The SHINE Trial Infant Feeding Intervention: Pilot Study of Effects on Maternal Learning and Infant Diet Quality in Rural Zimbabwe** Desai et al. [49]	Zimbabwe -—-—-—- **CASP** Low	Evaluate the independent and compounded effects of improved WASH & infant feeding on child stunting and anaemia; infant feeding intervention was pilot-tested to asses comprehension of messages and tools, as well as feasibility of a VHW- delivered intervention	9 VHWs delivered programme to 19 mother-infant dyads Infants aged 7–12 months	Mixed methods; effectiveness evaluation, knowledge assessment, feasibility evaluation	**Intervention** All 4 treatment arms: VHWs make 15 visits to mother–infant dyads, providing specific health messages, with four visits on EBF promotion Intervention arms: VHWs provide lessons on WASH, IYCF, or WASH + IYCF, with specific lessons depending on intervention randomised to (WASH, IYCF, or WASH + IYCF) **Comparator** VHWs make 15 visits to mother–infant dyads, providing specific health messages, with four visits on EBF promotion, but no visits providing specific promotion of WASH, IYCF, or both	Maternal knowledge about infant feeding; self-reported nutrient consumption of children (24 h recall)	Maternal knowledge on infant feeding improved after the dissemination of each lesson; responses showed knowledge absorption and retention on important feeding practices; consumption of each nutrient taught about and measured increased significantly; all infants received adequate vitamin A & fat; most infants consumed sufficient daily energy (79%), protein (95%), calcium (89%), & zinc (89%); percentage of infants achieving folate requirement was only 68%, though this was double the previous percentage. Infants reaching iron requirement increased from 0 to 68%	Stunting, micronutrient deficiencies	
**Health Extension Workers’ diagnostic accuracy for common childhood illnesses in four regions of Ethiopia: a cross‐sectional study** Getachew et al. [42]	Ethiopia -—-—-—- **CASP** Moderate	Investigate HEWs' ability to correctly diagnose childhood illnesses of diarrhoea, febrile disorders, acute respiratory tract infection, malnutrition, & ear infection	186 HEWs; 620 children 2–59 months observed and re-examined	Cross-sectional survey; observations of HEWs’ diagnosis were followed by a re-examination of the child by a trained health officer	ICCM delivered by HEWs; assessment, classification, and diagnosis of childhood illnesses by HEWs	HEW ability to correctly identify and classify childhood illnesses **Malnutrition diagnostics:** WFH/L, oedema, MUAC, medical complications, ability to finish RUTF for children older than 6 months, existence of breastfeeding issue for children younger than 6 months	**Key findings:** More research is needed on whether HEWs accurately assess and classify childhood illnesses; study results suggest a significant number of sick children were not correctly diagnosed, which could lead to lack of or incorrect treatment; efforts are needed to improve HEWs’ diagnostic ability for childhood illnesses and their adherence to the guidelines for the examination, classification and treatment of childhood illnesses Diagnosis by HEWs had a 39% & specificity 99% for malnutrition	Acute malnutrition: MAM, uncomplicated SAM, complicated SAM defined with WHO growth standards	
**Effect of complementary feeding behaviour change communication delivered through community‐level actors on infant growth and morbidity in rural communities of West Gojjam Zone, Northwest Ethiopia: A cluster‐randomized controlled trial** Ayalew et al. [33]	Ethiopia -—-—-—- **CASP** Low	Investigate effect of complementary feeding BCC disseminated through community-level actors on infant morbidity & growth	Children under 6 months at start of trial Baseline total: 612 (N-intervention: 306, N-control: 306); Follow-up total: 554 (N-intervention: 272, N-control: 282); Sample size total: 612 (N-intervention: 306, N-control: 306)	Cluster-randomized control trial	**Intervention** Infants, caregivers of infants, & family members in intervention clusters received complementary feeding BCC from community-level actors for 9 months. 3-part intervention: training of women development army (WDA) leaders; group training of mothers by WDA leaders; home visits **Comparator** Infants, caregivers of infants, & family members in control clusters received standard health and nutrition care	Effect on infant growth (stunting, underweight, wasting); effect on infant morbidity	**Key findings:** complementary feeding BCC disseminated by community-level actors significantly improved gains in infant length & weight. It also decreased rates of stunting & underweight Intervention infants had significantly higher weight gain (MD: 0.46 kg; 95% CI 0.36–0.56) and length gain (MD: 0.96 cm; 95% CI 0.56–1.36) versus control infants. The intervention infants also had a reduced rate of stunting by 7.5 percentage points (26.5% vs. 34%, RR = 0.68; 95% CI 0.47–0.98) and underweight by 8.2 percentage points (17% vs. 25.2%; RR = 0.55; 95% CI 0.35–0.87)	Stunting (HAZ < −2), underweight (WAZ < −2), wasting (WHZ < −2)	**Facilitators:** ability to influence change and feeding practices; intervention targeted family members in addition to mothers of infants—influencing the overall household environment to encourage change in behaviour; cooking demonstrations
**Précis of nutrition of children and women in Haiti: analyses of data from 1995 to 2012** Ayoya et a. [51]	Haiti -—-—-—- **CASP** Low	Address the information gap for nutrition issues in Haiti, research: trends and determinants of IYCF practices; micronutrient deficiencies; status of SAM in children; links between women’s empowerment, healthcare access, WASH, & child nutrition; community-based child nutrition initiatives; & nutrition governance status	Not specified	Mixed methods: secondary data analysis of national data sets; household survey; site visits; stakeholder interviews; document review; multivariate analyses to distinguish relationships between potential determinants of primary outcomes; baseline surveys; literature review	**Intervention** IYCF counselling; dispensation of MNPs to fortify complementary foods at home; and CMAM for children under 5 and pregnant and lactating women; GMC; vaccinating children; vitamin A for children, and deworming for children under 5 years old		CHW role is key to achieving an integrated health system; CHW role is key to achieve integrated health system; CHWs with substantial institutional support were among the most motivated and dedicated team members; CHWs played an essential role in screening and follow-up for services not delivered at the community level; numerous delivery platforms (including BCC) are required to implement a comprehensive nutrition programme; engaging community members in programme delivery can foster community involvement and encourage peer support for behaviour change	Micronutrient deficiencies, SAM, acute malnutrition	**Facilitators:** ability to ensure children in catchment area access all available services; strong institutional support; foster community engagement and peer support for behaviour change
Community volunteers can improve breastfeeding among children under six months of age in the Democratic Republic of Congo crisis Balaluka et al.[44]	DRC -—-—-—- **CASP** Moderate	Assess the effectiveness of CHWs in encouraging EBF from birth in a context of endemic malnutrition	Children under 6 months old Intervention: 208 children Comparator: 178 children	Cohort study; impact evaluation	**Intervention** Katana district: selected villages given team of 5 CHWs trained in promoting optimal breastfeeding practices. CHWs promoted EBF through door-to-door visits & community meetings. From 2004 to 2006, CHWs also helped supervise infant growth by arranging monthly community weighing sessions (supervised by district health officers) with a nutrition sensitisation to raise mothers' awareness about the importance of breastfeeding & EBF from birth to 6 months **Comparator** Walungu district: far apart and not adjoining Katana district. No community-based nutrition project or CHWs, no programme specifically about breastfeeding practices	Proportion of infants receiving EBF by age	**Length of EBF time from birth was higher in the intervention group (median, range):** Intervention was 6 months (2 to 7) versus comparator was 4 months (1 to 6) (*p *< 0.001) **Proportion of infants receiving EBF at 6 months old was high in the intervention group:** Intervention was 57.7% (95%: CI, 50.9 to 64.5) versus comparator 2.7% (95%: CI, 1.1 to 6.6) (*p *< 0.001)	Endemic malnutrition, GAM	**Facilitators:** level of education of CHWs; proximity of CHWs to breastfeeding mothers; CHWs driven by the knowledge and gravity of malnutrition in the area; community involvement and engagement
**Combined infant and young child feeding with small-quantity lipid-based nutrient supplementation is associated with a reduction in anemia but no changes in anthropometric status of young children from Katanga Province of the Democratic Republic of Congo: a quasi-experimental effectiveness study** Addo et al. [36]	DRC -—-—-—- **CASP** Moderate	Evaluate impact of IYCF–SQ-LNS intervention on anaemia & growth in children	2995 children aged 6–18 months	Cross-sectional comparison; quasi-experimental effectiveness design	**Interveniton** Enhanced IYCF package: standard IYCF package plus improved counselling on IYCF, daily SQ-LNS for infants 6–12 months, community-based nutrition education for mothers and pregnant women, & reinforced CHW role through enhanced training & community-based outreach counselling by CHWs on IYCF & SQ-LNS **Comparator** Standard IYCF package: iron–folic acid supplementation, antimalarial medication, individual IYCF counselling during ANC visits; individual counselling on IYCF & child health by CHWs during clinic visits; monthly group counselling on IYCF & child health at health clinics only; & IYCF counselling during HW outreach clinics	Anaemia prevalence; haemoglobin; iron levels, vitamin A levels; anthropometry measures (WAZ & LAZ); stunting prevalence	**Key findings:** Enhanced IYCF intervention was associated with a reduction in anaemia prevalence, increase in haemoglobin, but no effect on anthropometry or iron or vitamin A deficiencies. Intervention children who received ≥ 3 monthly SQ-LNS batch distributions had higher anthropometry measures and haemoglobin and lower prevalence of stunting than control children Enhanced IYCF intervention associated with 11.0% point (95% CI − 18.1, − 3.8; *P *< 0.01) adjusted relative reduction in anaemia prevalence and a mean + 0.26-g/dL (95% CI 0.04, 0.48; *P *= 0.02) increase in haemoglobin but no effect on anthropometry, iron, or vitamin A deficiencies Endline in the intervention: compared with those who didn’t receive any, children 8–13 months who received ≥ 3 monthly SQ-LNS batch distributions had higher anthropometry measures (LAZ: + 0.40, *P *= 0.04; WAZ: + 0.37, *P *= 0.04) and haemoglobin (+ 0.65 g/dL, *P *= 0.007) and a lower adjusted prevalence difference of stunting (− 16.7%, *P *= 0.03)	Stunting	
**Quality of care for treatment of uncomplicated severe acute malnutrition delivered by community health workers in a rural area of Mali** Alvarez Morán et al. [45]	Mali -—-—-—- **CASP** Moderate	Assess quality of care delivered by CHWs for uncomplicated SAM	17 CHWs; 125 cases of children under the age of 5 years old assessed and treated by CHWs	Observational, clinical prospective multicentre cohort study	**Interveniton** CHWs trained & equipped to treat uncomplicated SAM cases in children under 5 in the community. Pilot assessed effectiveness of treatment, measuring clinical outcomes, quality of care (technical competence) provided by CHWs, treatment coverage, & cost-effectiveness. Observers of CHWs were medical health professionals familiar with malnutrition treatment protocols **Comparator** Existing outpatient health facility treatment of SAM	**CHW quality of care** (capacity to evaluate, classify, & treat uncomplicated SAM; provide nutritional counselling to caretakers of children receiving treatment for SAM, malaria, pneumonia, or diarrhoea; & correctly refer complicated SAM)**; CHW technical competence** (screening for SAM; diagnosis of SAM; provision of antibiotics, vitamin A, and anti-parasitic medication for SAM; delivery of RUTF until child has recovered); **CHW interpersonal skills** (how CHW interacts with carer and child)	**Key findings:** 1) Well‐trained & supervised CHWs can manage uncomplicated SAM, including treatment and correct dosing with a high quality of care 2) Direct management of SAM cases by CHWs can enable increased access to quality SAM treatment in Mali & possibly other contexts 3) Need further research into resources required for continuous service delivery at the community level 100% of CHWs were observed as interacting correctly with patients & carers; 97.6% of children were correctly assessed for presence of cough, diarrhoea, fever, & vomiting; 78.4% oedema correctly assessed; 100% height measured correctly; 100% correctly classified for SAM; 77.8% correctly performed appetite test; 75% SAM cases administered correct medical treatment with Amoxicillin, Albendazole, & vitamin A; 94.3% of caretakers given essential nutrition counselling; 83.3% caretakers given demonstration on first treatment doses & correctly provided information on all treatments and dosage; 100% of cases assessed correctly administered RUTF; 79.5% of cases achieved composite indicator including all essential tasks to provide high-quality treatment for SAM (child appropriately assessed for key indicators, correctly classified & treated, and received key counselling)	Uncomplicated SAM in children under 5 years old defined by national protocaol as MUAC < 11.5 cm, WHZ < − 3, and/or nutritional oedema	**Barriers:** balancing increased workload when scaling up a programme **Facilitators:** supervision; good training
**The effectiveness of treatment for Severe Acute Malnutrition (SAM) delivered by community health workers compared to a traditional facility based model** Alvarez Morán et al. [46]	Mali -—-—-—- **CASP** Moderate	Investigate potential for integrating SAM identification & treatment delivered by CHWs to improve SAM treatment coverage	Children between 6–59 months old with SAM in neighbouring sectors of Kita district **Intervention:** 699 children **Control:** 235 children	Multicentre, randomised and rationalised intervention study	**Interveniton** Treatment for uncomplicated SAM from health centres or CHWs **Comparator** Outpatient treatment for uncomplicated SAM from health centres	**Clinical outcomes:** cure (child with WHZ ≥ −1.5 or MUAC > 125 mm and absence of nutritional oedema for 14 days), defaulter, & death ratios; **Other outcomes:** quality of care; treatment coverage (using the Semi-Quantitative Evaluation of Access and Coverage (SQUEAC) methodology)	**Key findings:** CHWs are effective in treating uncomplicated SAM in children & have non-inferior outcomes compared to traditional outpatient therapeutic feeding (OTP) treatment models. CHWs-delivered SAM treatment supported improved access to treatment Intervention cure ratio 94.2% compared to 88.6% in control (RR 1.07 [95% CI 1.01; 1.13]); defaulter ratios twice as high in control compared to intervention (10.8% vs 4.5%; RR 0.42 [95% CI 0.25; 0.71]); differences in mortality ratios not statistically significant (0.9% intervention compared to 0.8% control); coverage rates 86.7% intervention compared to 41.6% control (*p *< 0.0001)	Uncomplicated SAM in children defined by national protocol as children aged 6–59 months, MUAC < 115 cm, WHZ < − 3, and/or nutritional oedema	
**Reproductive, maternal, newborn and child health service delivery during conflict in Yemen: a case study** Tappis et al. [55]	Yemen -—-—-—- **CASP** Very low	Investigates how facility- and community-based RMNCAH + N services have been delivered since 2015, and factors influencing service implementation	3 governorates (Sana's City, Aden, Taiz); 181 individuals interviewed	Case study; content analysis methods for publicly available documents & datasets; 94 individual & group interviews with government officials, humanitarian agency staff, & facility-based healthcare providers; 6 FGDs with community health midwives & CHVs	**Intervention** Facility- and community-based RMNCAH + N services	Factors affecting RMNCAH + N service delivery, service availability, service coverage, and service quality	Humanitarian work and programmes centred on supporting and continuing the provision of basic services a facilities, & using mobile clinics, outreach teams, & CHVs to address emergency needs when the conflict environment allowed for movement & outreach to communities. The focus of specific sub-elements of RMNCAH + N depended on location, with these geographic changes due to differing priorities across different government offices or catchments, the level of active conflict, the ability to access affected populations; & qualified workforce availability; Overall, services for women’s health and child were prioritized. Otherwise, controlling cholera outbreaks & treatment of acute malnutrition were prioritized over other services	Acute malnutrition (MAM and SAM)	**Barriers:** Insecurity; resource-constraint of health facilities; challenges in importation distribution of supplies; politicization of aid; weak health system capacity; costs of care seeking; ongoing cholera epidemic; distrust & subsequent lack of demand **Facilitators:** Resilient healthcare workers
**Performance of low-literate community health workers treating severe acute malnutrition in South Sudan** Van Boetzelaer et al. [50]	South Sudan -—-—-—- **CASP** Moderate	Evaluate if low‐literate CBDs can adhere to a simplified SAM treatment protocol and to investigate the community acceptability of CBDs delivering treatment	57 CBDs; 141 performance checklists	Mixed methods pilot study; feasibility & acceptability study	**Intervention** CBDs trained and aided with tools adapted for low literacy as well as a simplified SAM treatment protocol. CBDs then returned to their communities where they passively screened for children suffering from uncomplicated SAM	Performance of the low‐literate CBDs in adhering to the treatment protocol	Low‐literate CBDs in South Sudan could adhere to a simplified treatment protocol for uncomplicated SAM through the use of low-literacy adapted tools. The number of performance checklists completed for a CBD was significantly associated with the last performance score recorded for the CBD. For each performance checklist completed, the final score of the CBD rose by absolute 2.0% (95% CI 0.3%–3.7%)	SAM	**Barriers:** High food insecurity & demand for RUTF led to conflict or suspicion in community around when a child was decided to not be eligible for treatment **Facilitators:** Manageable workload (SAM treatment provided on fixed day per week); community trust in CBDs; training: use of songs, practical exercises, role plays effective for training; supervision; proximity
**Sustainable under nutrition reduction program and dietary diversity among children’s aged 6–23 months, Northwest Ethiopia: Comparative cross-sectional study** Worku et al. [41]	Ethiopia -—-—-—- **CASP** Moderate	Compare level of dietary diversity among children aged 6–23 months in districts covered and not covered by the Sustainable Undernutrition Reduction in Ethiopia (SURE) programme in West Gojjam zone	832 mother–child pairs; Children aged 6–23 months Sample size: 832	Community-based, comparative cross-sectional study; mother and child pairs were selected by the simple random sampling technique. A pretested and structured interviewer-administered questionnaire was used to collect data. A binary logistic regression model was fitted to identify factors associated with dietary diversity. Crude odds and adjusted odds ratios with 95% CI calculated to assess the strength of associations and significance of the identified factors for dietary diversity score	**Intervention** SURE government-led, multi-sectoral programmes for the improvement of nutrition outcomes that particularly focuses on the integration of the health and agriculture sectors. It provided nutrition education using the BCC approaches. The project has three main components: enhancing community-based nutrition (CBN) to address inadequate complementary feeding, improving household dietary diversity through IYCF, and familiarising nutrition-sensitive agriculture **Comparator** Areas of similar demography not exposed to SURE programme	dietary diversity (number of different food groups consumed by the child in 24 h prior to assessment);	SURE programme covered districts 2.5 times more likely to have adequate dietary diversity than uncovered ones. The overall proportion of adequate dietary diversity among children aged 6–23 months was 29.9% (95% CI 27.0–33.0), whereas in SURE covered and uncovered districts it was 33.4% (95% CI 29.0–38.and 26.4%(95% CI 22.0, 31.0), respectively. ANC (Antenatal care) (AOR = 1.7; 95% CI 1.16, 2.55) and postnatal care services (AOR = 2.1; 95% CI 1.38, 3.28), participating in food preparation programmes (AOR = 1.9; 95% CI 1.19, 2.96), GMP (AOR = 2.74,95% CI 1.80, 4.18), vitamin A supplementation (AOR = 2.10,95% CI1.22, 3.61) and household visits by health extension workers (AOR = 2.0; 95% CI 1.25, 3.21) were significantly associated with dietary diversity	Undernutrition	**Facilitators:** women participating in food preparation programmes, household visits from HEWs, ANC visits, PNC follow-ups
**Cost-effectiveness of the treatment of uncomplicated severe acute malnutrition by community health workers compared to treatment provided at an outpatient facility in rural Mali** Rogers et al. [53]	Mali -—-—-—- **CASP** Moderate	Evaluate costs and cost-effectiveness of CHW-delivered care compared to outpatient facility-based care for SAM	Modelling number of children aged 6–59 months treated in each arm using sample size in intervention (n = 617); 18 CHWs; interviews (n = 59); FGDs (n = 10, 5 per arm); carers with a child in treatment or recently exited (n = 68)	Costs & cost-effectiveness assessment based off of a prospective multicentre clinical cohort trial conducted to evaluate treatment of uncomplicated SAM by CHWs versus existing outpatient facility-based care	**Intervention** 18 CHWs screening for SAM, making referrals to health clinics for complicated cases, & treating uncomplicated cases in the community; CHWs delivered nutrition sensitisations to communities. Alongside the CHW programme, 3 outpatient health facilities managed SAM cases **Comparator** 16 CHWs screened & delivered nutrition education sessions. They adhered to the Malian CMAM protocol in place at the time, and thus referred all cases to the outpatient facility for treatment or further referral to an inpatient care facility	Costs & cost-effectiveness	Costs were higher in the intervention than the control. CHW costs in the intervention arm were close to 3 × higher than the control, due to the greater amount of labour and involvement from CHWs in delivering services. Beneficiary costs were higher in the intervention group as a result of higher enrolment in the programme. However, at the individual household level, intervention households spent less time and money receiving treatment than control households. The base case analysis indicates outpatient facility-based care is more expensive than CHW-delivered care, both for providers and for beneficiaries. CHW-delivered care households spent almost half the time receiving treatment and 3 × less money compared with the outpatient facility-based arm (2.15 h versus 3.92 h; 0.60 USD versus 1.70 USD). Higher costs and time spent were attributed to transportation to the facility. Cost-effectiveness in the base case with the observed number of children treated, the average cost per child treated by CHWs was 244 USD compared to 442 USD in the outpatient facility. The cost per child recovered was 259 USD by CHWs and 501 USD in the outpatient facility	Uncomplicated SAM	**Facilitators:** Cost effective, lower beneficiary costs due to lower transport costs and time; lower provider costs in CHW arm
**Lipid-Based Nutrient Supplementation Reduces Child Anemia and Increases Micronutrient Status in Madagascar: A Multiarm Cluster-Randomized Controlled Trial** Stewart et al. [31]	Madagascar -—-—-—- **CASP** Moderate	Determine the effectiveness of LNS supplementation administered daily on child anaemia and micronutrient status within the context of an nutrition programme that is already running and has been scaled up	125 communities with children aged 0–24 months	Multiarm cluster-RCT	**Intervention** Treatment arms: (T1): T0 + home visits for intensive nutrition counselling (with additional CNWs); (T2): T1 + LNS for children aged 6–18 months (with & distributed by CNWs); (T3): T2 + LNS for pregnant and lactating women; (T4): T1 + early childhood stimulation and parenting messages **Comparator** (T0): Status quo treatment arm: based on standard Madagascan growth monitoring and nutrition education protocol Key messages: maternal nutrition, early initiation of breastfeeding, EBF for the first 6 months, continued breastfeeding through 2 years, & age-appropriate complementary feeding & hygiene behaviours. CNWs demonstrated cooking with local ingredients that were complementary foods. The government also distributed vitamin A biannually for children < 5 years old. Pregnant women were also given iron–folic acid supplements during ANC visits	Haemoglobin; anaemia; iron status; vitamin A status; all analyses were intention-to-treat	Children in the LNS groups (T2 and T3) had an approximately 40% lower prevalence of anaemia, 25% lower prevalence of iron deficiency than children in the control group (T0) (*P *< 0.05 for all). There were no differences in any of the biomarkers when comparing children in the T4 group with those in T0; nor were there differences between T3 and T2	Micronutrient deficiencies	
**Supplementary Feeding of Moderately Wasted Children in Sierra Leone Reduces Severe Acute Malnutrition and Death When Compared with Nutrition Counseling: A Retrospective Cohort Study** Rajabi et al. [43]	Sierra Leone -—-—-—- **CASP** High	Investigate if supplementary feeding versus counselling alone for children with moderate wasting was able to prevent progression to SAM or death	1791 children under 5 years old; sample size: 1092	Retrospective dual cohort study; 1791 children with moderate wasting taken from 2 RCTs that had occurred in the same location; 1077 children received supplementary feeding; 714 children received counselling only; children in both RCTs were followed for ≥ 24 weeks from enrolment	**Intervention** Supplementary feeding cohort was taken from a cluster-RCT (called FFS trial) comparing 4 different foods in the treatment of moderate wasting **Comparator** Counselling alone cohort taken from a cluster-RCT (called Hi-MAM trial) testing effectiveness of giving supplementary feeding and amoxicillin to higher-risk children with moderate wasting compared with counselling alone. Caretakers participated in mother support groups twice a week delivered by a trained community respected elder for 4 sessions which covered IYCF, cooking demonstrations, WASH, health care seeking, child development, & MUAC training for mothers	**Primary outcome:** time to SAM or death; SAM defined by MUAC < 11.5 cm and/or development of bilateral pedal pitting oedema **Secondary outcomes:** proportions of children with healthy MUAC, moderate wasting, SAM, and death; rates of gain in weight, MUAC, and length at 3 time points after enrolment: 6 weeks, 12 weeks (range: 8–18 weeks), and 24 weeks (range: 20–30 weeks)	In the counselling group (Hi-MAM), 47% attended all sessions and only 12% missed more than 1 session. Intervention children had a lower risk of developing SAM or dying over 24 weeks of follow-up, as well as greater rates of gain in weight and MUAC. For the entire follow-up period, children who received supplementary feeding were less likely to develop SAM or die (HR: 0.53; 95% CI 0.44, 0.65; *P *< 0.001). Children who received supplementary feeding were more likely to have a healthy MUAC at 6 & 12 weeks. They were also less likely to develop SAM at 6, 12, & 24 weeks & had higher rates of weight gain and MUAC gain at 6 & 12 weeks	Moderate wasting; SAM	**Facilitators:** Integration with or inclusion of supplementary feeding element
**Assessing the Impact of Integrated Community-Based Management of Severe Wasting Programmes in Conflict-Stricken South Sudan: A Multi-Dimensional Approach to Scalability of Nutrition Emergency Response Programmes** Renzaho et al. [52]	South Sudan -—-—-—- **CASP** Moderate	Analyse & report best practices & identify evidence on the effectiveness & scalability of CMSW programmes to support future nutrition interventions in South Sudan	1,105,546 children admitted to CMSW programmes over period of 5 years; targeted children under 5 years old, but still admitted older children with severe wasting	Multi-dimensional approach to assess impact, scalability, integration used to assess CMSW programmes’ impact. Used three data sources: standardised monitoring and assessment of relief and transitions (SMART), food security and nutrition monitoring system (FSNMS) surveys, & CMSW programmes’ performance data	Community-based management of severe wasting (CMSW) programmes	**CMSW Programme Scalability**: harmonisation of implementation; delivery system; technical assistance; organisational capacity; development & sharing of M&E evidence to guide policy & programmes; community ownership; partnership facilitation & coordination; defining of roles & responsibilities; financial resources & sustainability	Findings suggest strong CMSW programme implementation was associated with a timely manner and with quality care through an integrated, harmonised, multi-agency, and multidisciplinary approach. Between 2014 and 2019, wasting prevalence fluctuated with agriculture seasonality, remaining above the 15% emergency threshold during the lean season. But during the same period, under-five and crude mortality rates (10,000/day) declined respectively from 1.17 and 1.00 to 0.57 and 0.55. These two indicators remained below the emergency thresholds, suggesting emergency response was effectively managed. Over a five-year period, 1,105,546 children were enrolled into to CMSW programmes. The pooled performance indicators were as follows: 86.4 (18.9%) for recovery, 2.1 (7.8%) for deaths, 5.2 (10.3%) for defaulting, 1.7 (5.7%) for non-recovery, 4.6 (13.5%) for medical transfers, 2.2 (4.7%) for relapse, 3.3 (15.0) g/kg/day for weight gain velocity, and 6.7 (3.7) weeks for the length of stay in the programme. All key performance indicators, except the weight gain velocity, met or exceeded the Humanitarian Charter and Minimum Standards in Humanitarian Response **CMSW Performance:** Compared CMSW programme outcomes with SPHERE minimum standards using the following indicators: recovered, died, defaulted, medical transfers, not recovered, relapse, weight gain velocity, length of stay	Severe wasting	**Barriers:** Weak community mobilisation; poor context-specificity; insecurity & active conflict; resource constraints; reliance on imported RUTF emergency funding, & external assistance; weak health system; limited integration of programmes into public health systems; few opportunities for learning & knowledge transfer **Facilitators:** Development of comprehensive national protocols & standardisation of programme implementation; training & education for primary caregivers, government staff, NGO partner staff, and health workers on IYCF; multi-agency technical assistance & coordination; integrated, harmonised, & multidisciplinary programme & policy

**Table 4:** Summary table of study interventions

	Ability	Acceptability and feasibility	Effectiveness	Costs/cost-effectiveness	Key barriers	Key facilitators
Identifying AM			CVs identifying children highly susceptible to AM (Bisimwa et al.) [38]	Community-based screening by CHWs for AM (Isanaka et al.) [54]	CHWs screening for and treating uncomplicated SAM defined by national protocol as MUAC < 11.5 cm, WHZ < −3, and/or nutritional oedema (Alvarez Moran et al., 2017) [45]	CHWs screening for and treating uncomplicated SAM defined by national protocol as MUAC < 11.5 cm, WHZ < −3, and/or nutritional oedema (Alvarez Moran et al., 2017) [45]
			HEWs diagnosing MAM, uncomplicated SAM, and complicated SAM using WHO growth standards and definitions (Getachew et al.) [42]			
			CHWs screening for and treating uncomplicated SAM defined by national protocol as MUAC < 11.5 cm, WHZ < −3, and/or nutritional oedema (Alvarez Moran et al., 2017) [45]			
			CHWs screening and following up for malnutrition services (Ayoya et al., 2012) [51]			
Monitoring AM		cGMP programme delivered by illiterate CHWs (Mayhew et al.) [40]	CVs monitoring the growth of children through conducting community weighting sessions (Bisimwa et al.) [38]		CVs monitoring the growth of children through conducting community weighting sessions (Bisimwa et al.) [38]	CVs monitoring the growth of children through conducting community weighting sessions (Bisimwa et al.) [38]
Managing severe wasting					Community-based management of severe wasting (CMSW) (Renzaho et al.) [52]	Community-based management of severe wasting (CMSW) (Renzaho et al.) [52]
Treating AM	CHWs treating uncomplicated SAM defined as MUAC < 11.5 cm, WHZ < −3, and/or nutritional oedema (Alvarez Moran et al., 2017) [45]	CBDs adhering to a simplified SAM treatment protocol for uncomplicated SAM (Van Boetzelaer et al.) [50]	CHWs screening for and treating uncomplicated SAM defined by national protocol as MUAC < 11.5 cm, WHZ < −3, and/or nutritional oedema (Alvarez Moran et al., 2017) [45]	CHW-delivered treatment for uncomplicated SAM (Rogers et al.) [53]	CHWs screening for and treating uncomplicated SAM defined by national protocol as MUAC < 11.5 cm, WHZ < −3, and/or nutritional oedema (Alvarez Moran et al., 2017) [45]	CHWs screening for and treating uncomplicated SAM defined by national protocol as MUAC < 11.5 cm, WHZ < −3, and/or nutritional oedema (Alvarez Moran et al., 2017) [45]
	CBDs adhering to a simplified SAM treatment protocol for uncomplicated SAM (Van Boetzelaer et al.) [50]		CHWs treating uncomplicated SAM as defined as children 6–59 months, MUAC < 115 cm, WHZ < − 3, and/or nutritional oedema (Alvarez Moran et al., 2018) [46]		CBDs adhering to a simplified SAM treatment protocol for uncomplicated SAM (Van Boetzelaer et al.) [50]	CBDs adhering to a simplified SAM treatment protocol for uncomplicated SAM (Van Boetzelaer et al.) [50]
	CHWs treating uncomplicated SAM with different levels of supervision (Charle-Cuéllar et al.) [47]				RMNCAH + N community-based services, including community-based referral and treatment of AM (Tappis et al.) [55]	CHW-delivered treatment for uncomplicated SAM (Rogers et al.) [53]
IYCF BCC		VHW-delivered BCC to mothers on EBF, IYCF, & WASH (Desai et al.) [49]	CHWs with enhanced IYCF training conducting outreach counselling on IYCF (Addo et al.) [36]		CHWs and community leaders delivering intensive, community-based BCC on IYCF (Kim et al.) [39]	CHV-led monthly nutrition sessions on breastfeeding, EBF, water treatment, and IYCF for poor women, attendance incentivized with cash (Kurdi et al.) [32]
			cGMP programme delivered by illiterate CHWs (Mayhew et al.) [40]			
			CHWs and community leaders delivering intensive, community-based BCC on IYCF (Kim et al.) [39]			CHWs delivering community-based, complementary feeding BCC (Ayalew et al.) [33]
			HEWs delivering a BCC programme on CBN, IYCF, and nutrition-sensitive agriculture (Worku et al.) [41]		cGMP programme delivered by illiterate CHWs (Mayhew et al.) [40]	cGMP programme delivered by illiterate CHWs (Mayhew et al.) [40]
			CHWs-delivered complementary feeding BCC (Ayalew et al.) [33]			HEW-delivered BCC programme on CBN, IYCF, and nutrition-sensitive agriculture (Worku et al.) [41]
			VHW-delivered BCC to mothers on EBF, IYCF, & WASH (Desai et al.) [49]			CHWs promoting EBF through door-to-door visits and community meetings (Balaluka et al.) [44]
			VHW-delivered BCC for complementary feeding and use of locally available complementary foods (Paul et al.) [48]			VHW-delivered BCC for complementary feeding and use of locally available complementary foods (Paul et al.) [48]
			CHV-led monthly nutrition sessions on breastfeeding, EBF, water treatment, and IYCF for poor women, attendance incentivized with cash (Kurdi et al.) [32]			VHW-delivered BCC for complementary feeding methods and use of locally availability complementary foods (Paul et al.) [48]
			CHWs promoting EBF through door-to-door visits and community meetings (Balaluka et al.) [44]			
IYCF BCC & supplementary feeding		VHW-delivered BCC for complementary feeding methods, use of locally availability complementary foods, and use of LNS (Paul et al.) [48]	Facility-based supplementary feeding, plus nutrition counselling on IYCF and cooking by community elders, for children with moderate wasting (Rajabi et al.) [43]		Enhanced IYCF programme including community- and facility-based counseling on WASH, SQ-LNS, and IYCF; SQ-LNS distributions; and additional investments in the CHW platform including improved supervision, training, resources, and role clarity. (Locks et al.) [37]	Enhanced IYCF programme including community- and facility-based counseling on WASH, SQ-LNS, and IYCF; SQ-LNS distributions; and additional investments in the CHW platform including improved supervision, training, resources, and role clarity. (Locks et al.) [37]
			RUTF provision, plus antibiotics, plus nutrition counselling on IYCF and cooking by community elders, for children with high-risk MAM (Lelijveld et al.) [35]			
			Status quo CNW BCC based on standard Madagascan growth monitoring and nutrition education protocol, plus intensive CNW-delivered nutrition counselling, plus CHW-distributed LNS to children, plus LNS to pregnant and lactating women, plus CHW-distributed early children stimulation and parenting messages (Stewart et al.) [31]			
			Enhanced IYCF programme including community- and facility-based counseling on WASH, SQ-LNS, and IYCF; SQ-LNS distributions; and additional investments in the CHW platform including improved supervision, training, resources, and role clarity. (Locks et al.) [37]			
			Integrated management of AM through mother peer-counseling care groups with nutrition counselling on-site and at home visits, treatment of MAM with fortified blended flour, and treatment of SAM with RUTF (Maust et al.) [34]			

## Abbreviations

AEW: Agriculture extension worker
AOR: Adjusted odds ratio
BCC: Behaviour change communication
CAFS: Conflict-affected or fragile setting
CASP: Critical Appraisal Skills Programme
CBD: Community-based distributor
CBTC: Community-based therapeutic care
CTC: Community therapeutic care
CI: Confidence interval
CF: Child feeding
CHW: Community health worker
CM: Community mobilisation
CMAM: Community-based management of acute malnutrition
CMSW: Community-based management of severe wasting
CNW: Community nutrition worker
CSB: Corn/soy blend
CV: Community volunteer
CHV: Community health volunteer
DALY: Disability-adjusted life year
DiD: Difference in differences
FAO: The Food and Agriculture Organization
FGD: Focus group discussion
GAM: Global acute malnutrition
GCM: Global chronic malnutrition
GMC: Growth monitoring and counselling
HAZ: Height-for-age z-score
HDTL: Health development team leader (cadre of CHWs in Ethiopia)
HEW: Health extension worker (cadre of CHWs in Ethiopia)
HFA: Height-for-age
HR-MAM: High-risk moderate acute malnutrition
ICCM: Integrated community case management
ICER: Incremental cost-effectiveness ratio
IEC: Information, education, and communication
IMAM: Integrated management of acute malnutrition
IYCF: Infant and young child feeding
LAZ: Length-for-age z-score
LFA: Length-for-age
LMICs: Low- and middle-income countries
LNS: Lipid-based nutrient supplements
LR-MAM: Low-risk moderate acute malnutrition
MAM: Moderate acute malnutrition
MD: Mean difference
MM: Mass media
MNP: Micronutrient powder
MUAC: Mid-upper arm circumference
OR: Odds ratio
OTP: Outpatient therapeutic feeding programme
RCT: Randomised controlled trial
RD: Risk difference
RR: Risk ratio
RUF: Ready-to-use food
RUSF: Ready-to-use supplementary food
RUTF: Ready-to-use therapeutic food
SAM: Severe acute malnutrition
SQ-LNS: Small-quantity lipid-based nutrient supplements
TFC: Therapeutic feeding centre
UHC: Universal health coverage
UNICEF: The United Nations International Children’s Emergency Fund
USD: US Dollar
VHW: Village health worker
WASH: Water, sanitation, and hygiene
WAZ: Weight-for-age z-score
WBG: The World Bank Group
WDA: Women development army (cadre of CHWs in Ethiopia)
WFA: Weight-for-age
WFH: Weight-for-height
WFP: The World Food Programme
WHO: The World Health Organization
WHZ: Weight-for-height z-score

## Footnotes

1 Acute malnutrition is subdivided into two types: severe acute malnutrition (SAM) and moderate acute malnutrition (MAM). MAM, also known as moderate wasting, is defined by a WHZ ≥ -3 and < -2 standard deviations (SD) of the WHO child growth standards median, a MUAC ≥ 115 mm and < 125 mm, or both. SAM, also known as severe wasting, is defined by a WFH z-score < -3 SD of the WHO child growth standards median, a MUAC < 115 mm, the presence of a bilateral pitting oedema, or a combination of the three. Within the term SAM, the terms marasmus (referring to children very thin for their height) and kwashiorkor (referring to the presence of oedema in malnourished children) have been absorbed. Global acute malnutrition (GAM) refers to the combination of MAM and SAM and is used to measure the nutritional status of a population and as a severity indicator in emergency settings [3].

2 Stunting is defined as chronic malnutrition, with a HAZ < −2 [3].

3 Underweight is defined by a low weight-for-age z-score (WAZ) <  −2 SD of the WHO child growth standards median. An underweight child may be stunted, wasted, or both [2].
